# Engineering of high-precision C-to-G base editors with expanded site selectivity and target compatibility

**DOI:** 10.1093/nar/gkaf717

**Published:** 2025-08-11

**Authors:** Zheng Li, Wei Zhao, Shaokang Li, Xi Luo, Yinghui Wei, Yuanyan Zhang, Zhengyan Ye, Shangpu Li, Ming Wang, Junjie Tan, Ralph Bock

**Affiliations:** Sanya lnstitute of Nanjing Agricultural University, State Key Laboratory of Crop Genetics & Germplasm Enhancement and Utilization, Province and Ministry Co-sponsored Collaborative Innovation Center for Modern Crop Production, Jiangsu Engineering Research Center for Plant Genome Editing, Nanjing Agricultural University, Nanjing 210095, China; Zhongshan Biological Breeding Laboratory, No. 50 Zhongling Street, Nanjing 210014, China; Sanya lnstitute of Nanjing Agricultural University, State Key Laboratory of Crop Genetics & Germplasm Enhancement and Utilization, Province and Ministry Co-sponsored Collaborative Innovation Center for Modern Crop Production, Jiangsu Engineering Research Center for Plant Genome Editing, Nanjing Agricultural University, Nanjing 210095, China; Zhongshan Biological Breeding Laboratory, No. 50 Zhongling Street, Nanjing 210014, China; Sanya lnstitute of Nanjing Agricultural University, State Key Laboratory of Crop Genetics & Germplasm Enhancement and Utilization, Province and Ministry Co-sponsored Collaborative Innovation Center for Modern Crop Production, Jiangsu Engineering Research Center for Plant Genome Editing, Nanjing Agricultural University, Nanjing 210095, China; Zhongshan Biological Breeding Laboratory, No. 50 Zhongling Street, Nanjing 210014, China; Sanya lnstitute of Nanjing Agricultural University, State Key Laboratory of Crop Genetics & Germplasm Enhancement and Utilization, Province and Ministry Co-sponsored Collaborative Innovation Center for Modern Crop Production, Jiangsu Engineering Research Center for Plant Genome Editing, Nanjing Agricultural University, Nanjing 210095, China; Zhongshan Biological Breeding Laboratory, No. 50 Zhongling Street, Nanjing 210014, China; International Joint Agriculture Research Center for Animal Bio-Breeding of Ministry of Agriculture and Rural Affairs, College of Animal Science and Technology, Northwest A&F University, Yangling, Shaanxi 712100, China; Sanya lnstitute of Nanjing Agricultural University, State Key Laboratory of Crop Genetics & Germplasm Enhancement and Utilization, Province and Ministry Co-sponsored Collaborative Innovation Center for Modern Crop Production, Jiangsu Engineering Research Center for Plant Genome Editing, Nanjing Agricultural University, Nanjing 210095, China; Zhongshan Biological Breeding Laboratory, No. 50 Zhongling Street, Nanjing 210014, China; Sanya lnstitute of Nanjing Agricultural University, State Key Laboratory of Crop Genetics & Germplasm Enhancement and Utilization, Province and Ministry Co-sponsored Collaborative Innovation Center for Modern Crop Production, Jiangsu Engineering Research Center for Plant Genome Editing, Nanjing Agricultural University, Nanjing 210095, China; Zhongshan Biological Breeding Laboratory, No. 50 Zhongling Street, Nanjing 210014, China; International Joint Agriculture Research Center for Animal Bio-Breeding of Ministry of Agriculture and Rural Affairs, College of Animal Science and Technology, Northwest A&F University, Yangling, Shaanxi 712100, China; Department of Plant Pathology, Nanjing Agricultural University, Nanjing 210095, China; Sanya lnstitute of Nanjing Agricultural University, State Key Laboratory of Crop Genetics & Germplasm Enhancement and Utilization, Province and Ministry Co-sponsored Collaborative Innovation Center for Modern Crop Production, Jiangsu Engineering Research Center for Plant Genome Editing, Nanjing Agricultural University, Nanjing 210095, China; Zhongshan Biological Breeding Laboratory, No. 50 Zhongling Street, Nanjing 210014, China; Max-Planck-Institut für Molekulare Pflanzenphysiologie, Am Mühlenberg 1, D-14476 Potsdam-Golm, Germany

## Abstract

Base editors (BEs) are powerful tools for single nucleotide substitutions without requiring DNA double-stranded breaks or donor templates. The development of C-to-G base editors (CGBEs) represents a significant advancement by enabling base transversions, thus expanding the range of genetic modifications beyond traditional transitions and facilitating a broader spectrum of (therapeutic) applications. However, current CGBEs suffer from limitations in their editing range, mostly modifying position 6 relative to the distal end of the PAM, and their editing efficiency depends on the sequence context. In this study, by systematic exploration of deaminases to construct CGBEs, we have identified PmCDA1-based CGBEs that preferentially edit position 3. Furthermore, we report that truncations of the CDA1 C-terminus significantly enhance C-to-G editing efficiency. Our CDA1Δ-CGBEs not only exhibit high precision but also display remarkable compatibility with diverse substrate sequence contexts. We also show that they can substantially reduce, or even eliminate, genome-wide off-target editing. Importantly, we demonstrate that the strategy of using truncated CDA1 variants to improve C-to-G editing is effective not only in yeast but also in human and rice cells. These enhanced C-to-G base editing tools hold great promise for a wide range of applications in gene therapy, precision breeding, and fundamental research.

## Graphical Abstract

**Graphical Abstract:**
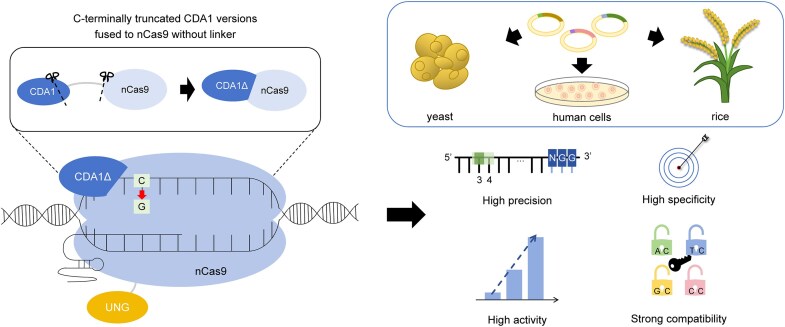

## Introduction

The CRISPR-Cas system functions as a bacterial adaptive immune system, protecting against invasive nucleic acids such as viruses and plasmids [1–5]. It consists of a guide RNA (gRNA) that identifies the target site via complementary base pairing and Cas proteins, typically comprising two nuclease domains that cleave both strands of the target DNA [2, 3, 6]. The Cas9 enzyme from *Streptococcus pyogenes* (SpCas9) is widely used for genome editing due to its successful repurposing for this application [2, 4, 7–10]. When the DNA is cleaved, double-strand breaks (DSBs) are generated, and subsequently fixed by cellular DNA repair mechanisms, typically non-homologous end joining (NHEJ). NHEJ is an efficient but error-prone pathway present in most cells, which can result in random insertions or deletions (indels) at the target sites, thus often leading to gene knockout by generation of loss-of-function alleles [6, 11].

CRISPR-Cas-based tools, despite of their effectiveness in producing gene knockouts, are still limited in their ability to efficiently and precisely introduce point mutations, which are required for gene therapy and precision breeding (e.g. >50% of hereditary human diseases arise due to point mutations) [12–14]. To address this issue, researchers have been working on re-engineering CRISPR-Cas systems for the introduction of sequence-specific DNA changes [15, 16]. One approach pursued has been to co-deliver Cas nucleases along with an exogenous DNA template that contains the desired genomic alterations and is flanked by homologous sequence to the target sites to activate the cellular homology-directed repair (HDR) pathway, a repair mechanism with high fidelity [17]. Although HDR allows a broad range of edits, its editing efficiency is typically low in most cell types. Moreover, HDR requires provision of donor DNA as a repair template, which also can be utilized erroneously by other repair pathways such as NHEJ, thus creating unintended mutations [6, 9, 11, 18, 19].

Since 2016, CRISPR base editors (BEs) have been developed, which involve the fusion of a catalytically impaired Cas nuclease with a nucleoside deaminase enzyme [20–23]. This allows for the introduction of point mutations without requiring a donor DNA template and without causing a DSB in the target DNA. Current BEs primarily achieve base transitions within the same group of nucleotides (i.e. either pyrimidine-to-pyrimidine or purine-to-purine substitutions) [6, 24]. For example, C-to-T substitutions can be accomplished using cytosine base editors (CBEs) [20, 21, 25, 26], while A-to-G conversions are made with adenine base editors (ABEs) [22]. Recently, a new type of BE, known as the C-to-G base editor (CGBE), has been established, comprising a Cas9 nickase (nCas9), a deaminase enzyme, and sometimes an additional uracil DNA N-glycosylase (UNG). Specificity is achieved through the precise pairing of the 20-nucleotide protospacer sequence of the gRNA with the complementary target DNA, forming an R-loop structure, followed by the deaminase enzyme converting cytosine to uracil on the non-target strand, which is then processed to generate the desired C-to-G base change [27–30]. Although significant efforts have been made to improve the editing efficiency and product purity (i.e. the proportion of all edited alleles containing only the desired edit) through deaminase engineering and/or fusion to extra base excision repair (BER) proteins [27–29, 31–33], currently available CGBEs suffer from serious limitations in their editing range as they selectively edit position 6 within the protospacer relative to the distal end of the protospacer adjacent motif (PAM) [27]. Also, their effectiveness heavily relies on the sequence context of the DNA substrates, exhibiting a strong editing preference for AT-rich sequence contexts [27, 29, 30], which severely restricts the range of potential applications for CGBEs.

In this study, we aimed to address the major limitations of current CGBEs by producing a more versatile set of CGBEs with an alternative editing window and improved target compatibility. To this end, we tested fusions of several deaminases (and variants of them) with the nCas9 and identified CGBEs based on PmCDA1 (hereafter referred to as CDA1) as exhibiting an alternative editing window. The CDA1 CGBE shows with the highest editing efficiency at position 3 relative to the distal end of the PAM. Furthermore, by C-terminal truncation of the CDA1 moiety within the CGBEs, we were able to further enhance editing efficiency and product purity for C-to-G editing. Our research also demonstrates that our new CDA1 CGBEs can achieve highly precise C-to-G editing, while exhibiting compatibility with a wide range of substrate sequences. These new and highly precise CGBEs significantly expand the possibilities of C-to-G editing and offer significant potential for applications in gene therapy, precision breeding, and fundamental research.

## Materials and methods

### Yeast strains and growth conditions

To facilitate the comprehensive evaluation of base editing efficiencies in a genetic background that is common in plants and animals, genomic editing assays in this research were conducted using the diploid yeast strain *Saccharomyces cerevisiae* BY4743 (*MAT* a/α, *his3*Δ*1/his3*Δ*1*,*leu2*Δ*0/leu2*Δ*0*,*LYS2/lys2*Δ*0*,*met15*Δ*0/MET15*,*ura3*Δ*0/ura3*Δ). The strain was propagated using YPAD non-selective liquid culture medium, consisting of 2% peptone, 1% yeast extract, 2% glucose, and 0.003% adenine hemisulfate, with the addition of 1.5% agar for plate cultures. Positive selection following transformation was achieved by identifying colonies that had successfully incorporated the desired genetic modification using the two-deficiency medium SC-L-U, containing 0.67% yeast nitrogen base (YNB), 2% glucose, and a mixture of the appropriate amino acids, excluding uracil and leucine. *URA3* and *LEU2* genes served as markers. To induce BE expression, the yeast strains were cultured in an induced liquid medium at 28°C, containing 0.67% YNB, 2% galactose, 1% raffinose, and a mixture of the appropriate amino acids, excluding uracil and leucine. The cultures were incubated on a rotary shaker at 225 rpm.

### DNA methods

Polymerase chain reaction (PCR) was performed with Phanta Max Super-Fidelity DNA Polymerase (Vazyme Biotech) following the manufacturer’s instructions. All primers used in this study are listed in Supplementary Tables S2-S4. To generate the BE R33A-BE3 for yeast, the point mutation R33A was introduced into rAPOBEC1 within the BE3 construct (pJT25; Addgene no. 143736) using primers containing the desired mutation. The *UNG1* gene utilized in our experiments originated from *S. cerevisiae* (*UNG1*, UniProtKB-P12887). The *rXRCC1* (UniProtKB-Q9ESZ0) gene was obtained as yeast codon-optimized *de novo* synthesized gene fragment (Tsingke Biotech) and cloned into the AscI/MluI-digested vectors CDA1(Δ194)-nCas9 and CDA1(Δ194)-nCas9-UNG1, respectively. All gRNA target sites investigated in this study are listed in Supplementary Table S1. To construct plasmids expressing single guide RNAs (sgRNAs) that target specific sites, protospacer sequences were introduced through PCR amplification (as part of the primer sequence; see Supplementary Table S2), and the resulting fragments were cloned into the AatII/KpnI-digested parental vector pJT303 (Addgene no. 145066) using the OK Clon Kit (Accurate, Hunan, P.R. China). To generate the BEs DAF-CBE and CE-CDG, CDG4 and hCDG, codon-optimized for yeast, were synthesized and subsequently cloned into the SpeI/SbfI-digested and SpeI/AscI-digested CDA1-miniCGBE vectors using the OK Clon Kit. All plasmids used for yeast transformation experiments were subjected to Sanger sequencing to verify their sequences, and were prepared using the Plasmid Mini or Midi Kits (Omega).

The BE plasmids for human cells and the sgRNA-encoding sequences were synthesized by HuaGene Co., Ltd., and cloned to generate constructs h-CDA1-CGBE and h-CDA1(Δ194)-CGBE using the pEASY^®^-Basic Seamless Cloning and Assembly Kit from TransGen Biotech. The sgRNA oligonucleotides were annealed and inserted into the Eco31I site of the constructs. For base editing in rice, the DNA sequences were codon-optimized for rice, synthesized by Beijing Tsingke Biotech Co., Ltd., and cloned into AvrII- and SbfI-digested pH-A3A-PBE vectors (Addgene no. 119774) to create the plasmid constructs p-CDA1-CGBE and p-CDA1(Δ194)-CGBE using the OK Clon Kit (Accurate, Hunan, P.R. China). DNA fragments for the sgRNA spacer sequences were produced by annealing synthetic oligonucleotides and then inserted into the BsaI site of the constructs via Golden Gate Assembly. The specific sgRNA spacer sequences are provided in Supplementary Table S1.

### Rice transformation

The BE constructs for rice were introduced into *Agrobacterium tumefaciens* strain EHA105 and subsequently transformed into embryogenic calli derived from mature seeds of the rice variety Ningjing 7 (*Oryza sativa* L. ssp. japonica). Three days after transformation, the calli were transferred to N6 medium supplemented with 2.5 mg/l 2,4-dichlorophenoxyacetic acid, 0.3 g/l casamino acids, 4.6 g/l Gelrite, 30 g/l sucrose, 0.25 g/l carbenicillin, and 75 mg/l hygromycin, and cultured for 30 days. Hygromycin-resistant calli were collected and pooled for genomic DNA extraction. The extracted DNA was subsequently genotyped using next-generation sequencing (NGS).

### Yeast transformation and genomic DNA extraction

Yeast cells were transformed using a previously described protocol [34]. In brief, cells were initially cultured on YPAD plates for 2 days. Freshly grown cells (25 μl per transformation) were collected, washed with sterilized ddH_2_O, and then incubated with 100 mM LiAc for 10 min. The cells were then incubated at 42°C with plasmid DNA mixtures containing 0.5-1 μg of plasmid DNA, 240 μl of 50% PEG3350, 36 μl of 1M LiAc, 50 μl of 2 mg/ml carrier DNA, and 20 μl of ddH_2_O, for a duration of 1-3 h. Transformed clones were selected on SC-L-U medium and their presence was confirmed by PCR analyses. Yeast genomic DNA was extracted following a published protocol [35]. PCR products were purified using the Cycle Pure Kit (Omega) and subsequently subjected to sequencing.

### Galactose induction of base editors

To initiate yeast cultures, three to five positive colonies were selected and suspended in 3 ml of SC-L-U medium containing 2% glucose. The cultures were then grown until they reached the stationary phase. Afterward, samples of 0.8 ml were pelleted, washed three times with sterile water to eliminate any remaining glucose, and resuspended in 5 ml of SC-L-U induction medium supplemented with 2% galactose and 1% raffinose, to achieve an OD_600_ of ∼0.3. The cells were then incubated under shaking conditions at 225 rpm for 20 h.

### Mammalian cell culture, transfection, and flow cytometry analysis

HEK293T cells were cultured in Dulbecco’s modified Eagle’s medium (Gibco) containing 10% fetal bovine serum (Gibco) and 1% penicillin-streptomycin (Gibco), in a humidified incubator at 37°C with 5% CO_2_. Cells were seeded at a density of 1 × 10^5^ per well in 24-well plates (Corning) and transfected at ∼80% confluence with CGBE expression plasmids (1500 ng) using 3 μl of polyethylenimine (PEI) at a 1:2 DNA (μg) to PEI (μl) ratio. After 60-72 h, cells were harvested and 0.25% trypsin (Gibco) was added for fluorescence-activated cell sorting. All mCherry-positive cells were collected for genomic DNA extraction.

### High-throughput DNA sequencing and data analysis

For sample preparation for high-throughput sequencing, freshly transformed colonies were selected and grown in 3 ml of SC-L-U medium with 2% glucose until they reached the stationary phase. Next, a 0.8-ml sample of each culture was washed and resuspended in SC-L-U induction medium supplemented with 2% galactose and 1% raffinose to achieve an OD_600_ of ∼0.3. The cultures were then grown on a rotary shaker at 225 rpm at 28°C for 20 h. Genomic DNA was extracted from 0.5 ml samples of each culture and the regions targeted by base editing were PCR amplified using primer pairs containing index tags for multiplexing (Supplementary Table S3). PCR amplification was carried out with Phanta Max Super-Fidelity DNA Polymerase (Vazyme Biotech), followed by purification of the amplified fragments with the NucleoSpin Gel and PCR Clean-up Kit (Omega). Subsequently, the resulting PCR products, labeled with indices, were pooled in equal molar ratios.

The pooled index-labeled PCR products were then subjected to commercial PCR-free library construction, high-throughput sequencing, demultiplexing, and data processing (Bioss, Beijing, China). Sequencing was performed using an Illumina NovaSeq 6000 platform, obtaining paired-end reads with a read length of 150 nt for each side. On average, >100 000 reads were obtained for each sample. After data filtering, clean FASTQ files were analyzed using scripts available at https://github.com/zfcarpe/Cas9Sequencing. Specifically after obtaining paired-end reads from high-throughput sequencing, the reads were aligned against the reference sequence to identify the target editing sites. The editing efficiency was then calculated by determining the percentage of reads at each editing site that contained the desired base change (target edits) relative to the total number of reads at that position.

### Whole-genome sequencing for analysis of off-target editing

Yeast strains expressing a variety of BE constructs, including CDA1 CGBE and four truncated versions, along with an sgRNA targeting the *Can1* site, were initially cultured in SC-L-U medium containing 2% glucose. Subsequently, the cultures were transferred to induction medium supplemented with 2% galactose and 1% raffinose and then incubated for 20 h. After induction, suitable volumes of each culture were plated on YPAD or SC-Arg media supplemented with 60 μg/ml l-canavanine (Sigma-Aldrich) and incubated for 3 days to select for resistant colonies. From each plate, three colonies were randomly selected, suspended in YPAD medium, and incubated overnight. The resulting cultures were then combined in equal volumes and genomic DNA was extracted using the Genomic DNA Purification Kit (Solarbio, China) following the manufacturer’s instructions. The quality of the extracted DNA samples was evaluated and commercially available services were employed for library construction, high-throughput sequencing, and bioinformatics analysis of the raw data (Bioss, Beijing, China). DNA sequencing was performed using an Illumina NovaSeq 6000 platform.

### RNA editing analysis by RNA-seq

Yeast total RNA was extracted using the RNA Extraction Kit (Thermo Fisher Scientific). Subsequently, NGS library construction and sequencing were performed commercially on the Illumina Novaseq 6000 platform (Bokaisen Biotech, Beijing, China). The initial processing of raw sequencing data was conducted with fastp (version 0.12.4) [36] to eliminate low-quality reads. Single nucleotide polymorphism (SNP) calling followed the recommended GATK workflow. The reads were then mapped to the yeast reference genome (S288C) using the STAR software (version 2.5.2b) [37] and transformed into BAM files. The AddOrReplaceReadGroups, MarkDuplicates, and SplitNCigarReads functions within GATK (version 4.5.0.0) [38] were employed to assign reads to new read groups, identify duplicate reads, and split reads with “N” in CIGAR, respectively. GATK HaplotypeCaller was then utilized for SNP calling with a QUAL threshold set at >20. VCFtools (version 0.1.16) [39] was used to extract SNP/Indel variants from the VCF files, and the SNV/Indel data were separately analyzed in the R (v4.3.2) environment.

## Results

### CDA1-derived CGBEs exhibit an alternative editing window for C-to-G editing

**Figure 1.**
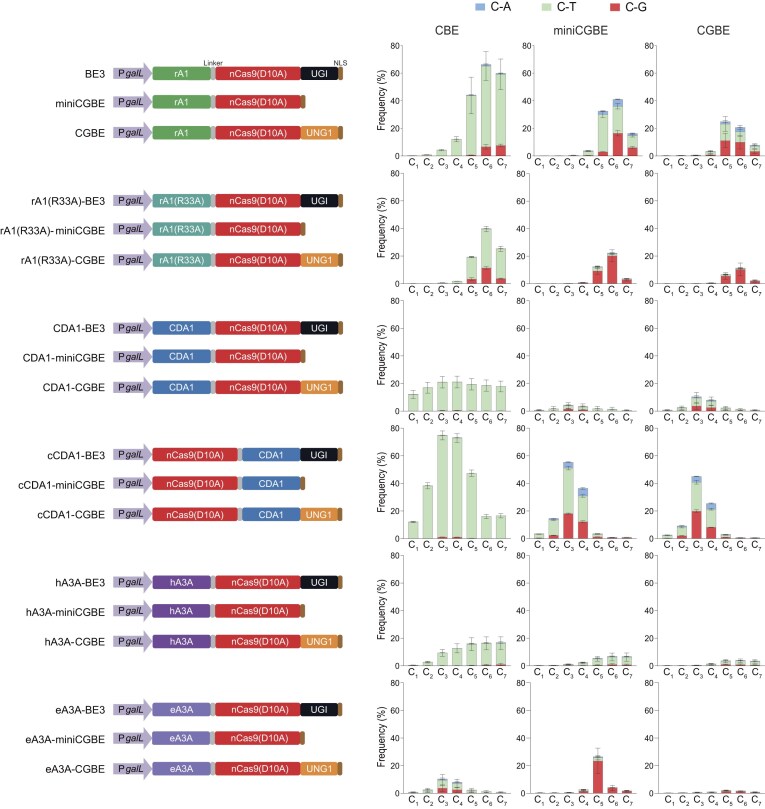
On-target DNA editing activities of various CBEs, miniCGBEs, and CGBEs. The left panel depicts the structures of the CBEs, miniCGBEs, and CGBEs tested. rA1: cytosine deaminase APOBEC1from *Rattus norvegicus*; rA1(R33A): the variant of rA1 that has R33A mutation; CDA1: cytosine deaminase CDA1 from *Petromyzon marinus*; cCDA1: C-terminal fusion of CDA1 to nCas9; hA3A: cytosine deaminase APOBEC3A from human cells; eA3A: engineered human APOBEC3A(N57G); nCas9 (D10A): *S. pyogenes* nCas9 with the mutation D10A; UGI: uracil-DNA glycosylase inhibitor; UNG1: uracil-DNA glycosylase from *S. cerevisiae*. The right panel displays the on-target editing frequencies of the different sets of CBE, miniCGBE, and CGBE (corresponding to the schematic maps in the left panel). The *x*-axis shows the Cs in the protospacer sequence with their position relative to the distal end of the PAM; the *y*-axis represents the percentage of total sequencing reads with the target C converted to other bases. Values and error bars represent the mean and standard deviation of three independent biological replicates. Source data are provided as a Source Data file in the Supplementary data.

Current CGBEs, typically based on APOBEC family proteins, predominantly edit nucleotides within the spacer sequence that are centered at position 6 (counting the PAM as 21-23) [27–30]. We previously showed that CDA1, an APOBEC homolog from sea lamprey, exhibits an alternative editing window with a pronounced editing preference for position 3 (relative to the distal end of the PAM), when replacing APOBEC proteins in cytosine BEs. Since CGBEs are CBE derivatives lacking the uracil glycosylase inhibitor (UGI), we hypothesized that CGBEs constructed with CDA1 could edit a different region than current APOBEC-based CGBEs, which would substantially broaden the applicability of C-to-G editing. To this end, we investigated the effect of three commonly used cytosine deaminases (and some of their variants) on base editing profile when combined with nCas9 and UNG (or solely fused to nCas9) to create sets of corresponding CBEs, miniCGBEs (without UNG), and CGBEs (with UNG). UNG1 from *S. cerevisiae* was used due to its reported superior performance over other UNG proteins [40]. The BEs were introduced into yeast (*S. cerevisiae*) cells to evaluate their base editing efficiencies on a polyC-containing target site, which serves as a stringent assay for evaluating site selectivity [26] (Fig. 1). While the BEs based on APOBEC deaminases [i.e. containing either rat APOBEC1 (rA1) or human APOBEC3A (hA3A); Fig. 1] showed the expected base editing window centered at position 5 or 6, the CDA1-based BEs, regardless of the nCas9 terminus to which CDA1 was fused, displayed an altered editing window with preferred editing occurring at position 3 in the protospacer (Fig. 1). Replacement of APOBEC deaminases with narrowed-window variants [i.e. rA1 (R33A) [41] and eA3A [42]] significantly increased C-to-G editing efficiency, consistent with previous reports [28, 29, 31]. In addition, we observed significant differences in editing efficiency between different CDA1 fusions. The C-terminal fusions (cCDA1-miniCGBE and -CGBE) exhibited notably higher levels of C-to-G editing than the N-terminal fusions (nCDA1-miniCGBE and -CGBE; Fig. 1). For CDA1-derived CBEs, the C-terminal fusion showed a more limited editing window (C_2_-C_5_) than the N-terminal fusion (C_1_-C_7_) [26] (Fig. 1). These findings may suggest that the conformational constraints that narrow the activity window of CBEs likely enhance the efficiency of C-to-G base editing.

### Engineering of improved CDA1-based CGBEs

**Figure 2.**
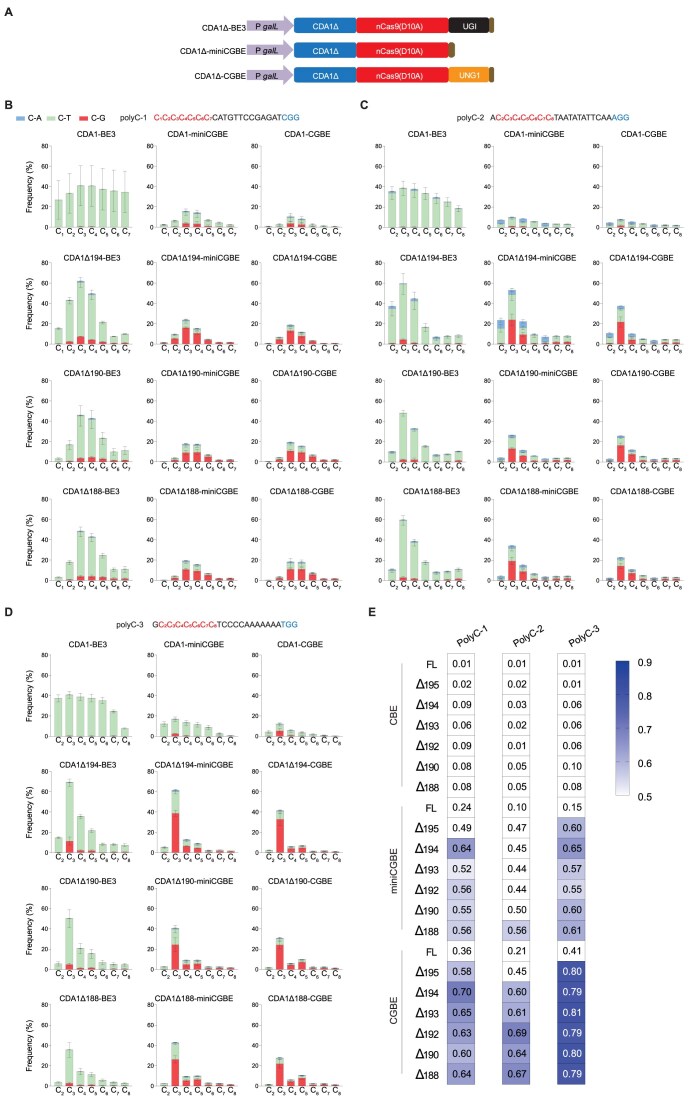
Improvement of C-to-G editing efficiency and purity with truncated CDA1 variants. (**A**) Structures of the CBEs, miniCGBEs, and CGBEs constructed with a series of truncated versions of CDA1. (**B**-**D**) Comparison of on-target DNA editing frequencies of CBEs, miniCGBEs, and CGBEs with CDA1 truncations at three polyC-containing sites. Values and error bars represent the mean and standard deviation of three biological replicates. Source data are provided as a Source Data file in the Supplementary data. For a comparison with additional deletion constructs, see Supplementary Fig. S1. (**E**) Heatmaps showing the on-target C-to-G editing purity of various BEs with C-terminally truncated CDA1 domains at the three target sites. The purity represents the percentage of edited sequencing reads (reads in which the target C has been converted to another base) in which the target C is edited to G. FL: full-length CDA1. Source data underlying panels (B)-(D) are provided as a Source Data file in the Supplementary data.

The outcome of the experiments described above indicated the feasibility of enhancing C-to-G base editing efficiency by engineering CGBEs via integration of deaminase domains that narrow down the editing window. Previously, we had demonstrated that C-terminal truncation of the CDA1 protein and its direct fusion to the N-terminus of nCas9 lead to a considerable reduction in the width of the base editing window of CBEs [26]. This reduction was attributed to a more narrowly defined distance between the nucleoside deaminase domain and the nCas9 domain of the fusion protein, likely resulting in more precise positioning of the deaminase domain on the target sequence [25, 26]. In the light of this, we conducted experiments with CGBEs and miniCGBEs, those with various truncated versions of CDA1, to assess their impact on efficiency and purity of C-to-G base editing. As deaminase domain, we employed the full-length CDA1 and different truncated versions lacking 13-20 amino acids from the C-terminus. Previous studies had shown that, when fused with nCas9, C-terminal deletions in this range confer the highest level of editing precision without compromising editing activity [25, 26]. We generated 14 CGBEs with C-terminally truncated CDA1 versions fused to nCas9, either with UNG1 (CDA1Δ-CGBEs) or without UNG1 (CDA1Δ-miniCGBEs), and evaluated their performance on three polyC motifs that provide stringent assays for the site selectivity of BEs [26] (Fig. 2A-D and Supplementary Fig. S1). For comparison, the corresponding CBEs were also included in these analyses.

While the full-length CDA1-based miniCGBE and CGBE exhibited remarkably low efficiency in C-to-G editing across all three target sites (whereas the corresponding CBEs displayed considerably higher C-to-T editing activity, as expected), the CDA1 truncations significantly improved the C-to-G editing efficiency. The truncated version CDA1Δ194 displaying the largest increase across the three target sites, ranging from 9.5- to 24-fold compared to the full-length version (Fig. 2 and Supplementary Fig. S1). Furthermore, we observed that CGBEs harboring UNG1, as opposed to miniCGBEs, effectively reduced C-to-T and C-to-A editing, while maintaining similar levels of C-to-G editing. Upon examining the editing purity of C-to-G at the three target sites, we observed that CGBEs exhibited significantly higher editing purity compared to miniCGBEs. For example, at the polyC-3 site, the editing purity reached ∼80% for CGBEs, but only 60% for miniCGBEs (Fig. 2E). We also investigated the indel frequencies at the three target sites following treatment with these BEs. This analysis indicated that both miniCGBEs and CGBEs induce a very low indel frequency (<5%), though slightly higher than that induced by the corresponding CBEs (Supplementary Fig. S2), consistent with recent reports [27]. Overall, these findings provide strong evidence for the effectiveness of engineered CGBEs with narrowed editing windows for precise and efficient C-to-G base editing.

### CDA1-based CGBEs achieve C-to-G editing with high precision

**Figure 3.**
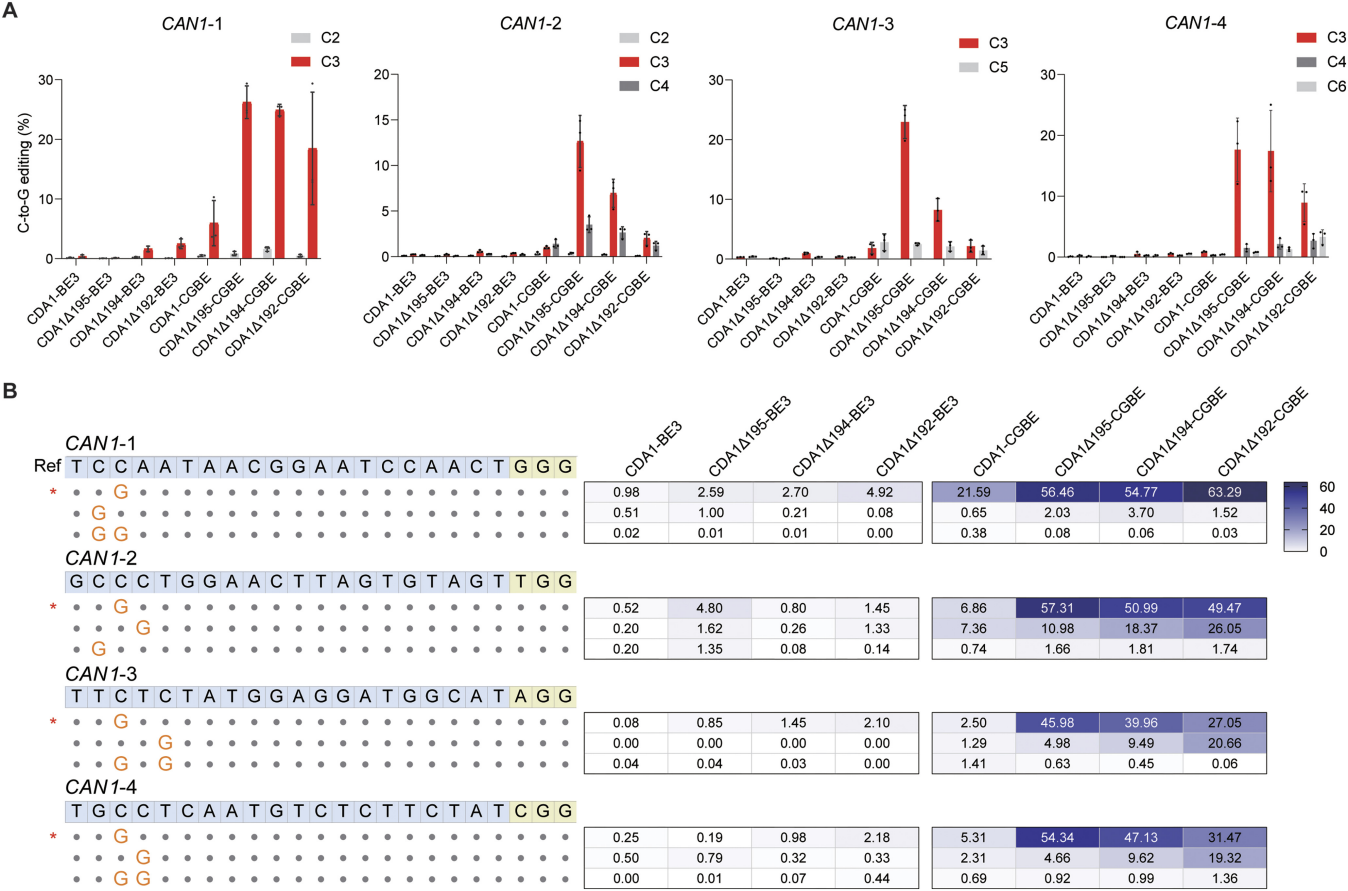
CGBEs with C-terminally truncated CDA1 domains edit position C_3_ with high precision. CDA1 CGBE, two selected CGBEs with C-terminally truncated CDA1 domains, and their CBE derivatives are compared. (**A**) Editing of four genomic loci containing multiple cytidines directly adjacent or in close proximity to C_3_. (**B**) CGBEs with truncated CDA1 domains significantly improve the purity of editing products and produce predominantly singly C_3_-modified products. Percentage of edited reads represents the proportion of sequencing reads that contained the edited products shown. Values and error bars represent the mean and standard deviation of three biological replicates. Source data underlying panels (A) and (B) are provided as a Source Data file in the Supplementary data. Ref: reference sequence.

Evaluation on oligo (C) motifs revealed that CDA1-based CGBEs exhibit highly precise editing, specifically targeting the C_3_ position relative to the distal end of the PAM. However, although providing rigorous test sequences (i.e. worst-case scenarios) for editing precision, long stretches of C nucleotides are not common targets of genome editing with BEs *in vivo*. To evaluate whether our new CDA1-based CGBEs with C-terminally truncated CDA1 domains also exhibit superior performance in more natural genomic sequence contexts, we selected four sites within the yeast *Can1* gene, each containing at least one additional C directly adjacent or in close proximity to the C_3_ position. Comparative analysis of the base editing outcomes with full-length CDA1 CGBEs, three truncated CDA1 CGBE versions and their corresponding CBEs revealed that CGBEs with CDA1 truncations exhibited significantly higher editing precision (Fig. 3). Across all four tested sites, CGBEs with CDA1 truncations predominantly edited the C_3_ position with up to 22-fold higher efficiency compared to other adjacent Cs (Fig. 3A). Upon examining the distribution of the editing products, it was observed that CGBEs with CDA1 truncations predominantly produced modified products with a single C-to-G modification at position 3, accounting for 45%-63% of all edited products (Fig. 3B). These findings provide evidence for superior precision and efficacy of CDA1-based CGBEs in achieving targeted C-to-G base editing.

### CDA1-based CGBEs display increased target context compatibility

**Figure 4.**
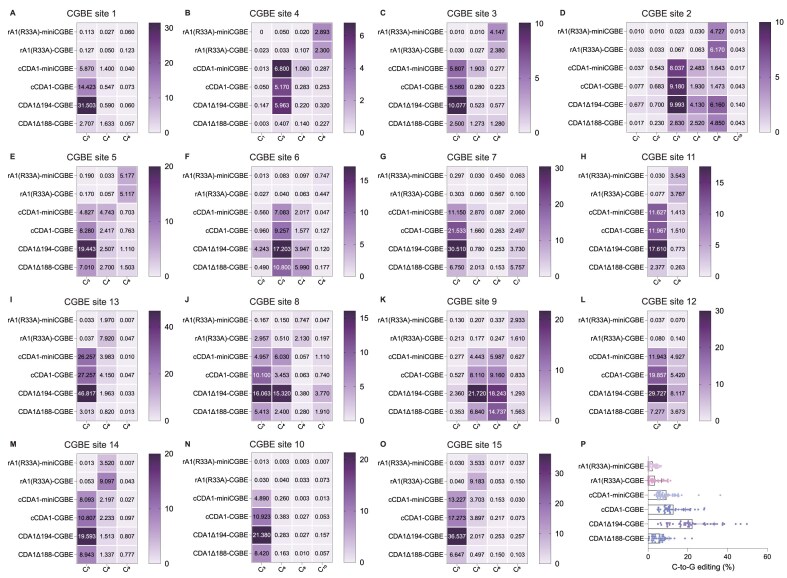
Comparison of base editing frequencies among CDA1-based CGBEs and rA1(R33A)-CGBE at 15 target sites. (**A**-**O**) Heatmaps displaying C-to-G editing frequencies at various cytosine positions across 15 target sites. Each heatmap represents the editing frequency for specific cytosine positions within the target sequences, with gradients ranging from light (indicating low editing frequency) to dark (indicating high editing frequency). Values inside each cell indicate the mean editing frequency (in %) of three independent biological replicates at the corresponding cytosine position. (**P**) Summary of C-to-G editing frequencies induced by the six CGBEs at 15 target sites. Each dot represents editing of an individual biological replicate. Source data are provided as a Source Data file in the Supplementary data.

Current CGBEs, primarily based on rat APOBEC1 (rA1) or its variant rA1 (R33A), display a strong dependence on substrate sequence contexts, with a pronounced preference for AT-rich sequence contexts [27, 31], which greatly restricts their applicability in a wide range of editing scenarios. In order to investigate the target context compatibility of our new CDA1-based CGBEs, the two truncated CDA1 versions, CDA1Δ194-CGBE and CDA1Δ188-CGBE, along with cCDA1-miniCGBE and cCDA1-CGBE (a fusion of CDA1 to the C-terminus of nCas9 with or without UNG1, both of which exhibited high editing activity; Fig. 1) were selected for evaluation of their editing capacities at 15 endogenous target sites that encompassed a wide spectrum of sequence contexts. For comparison, rA1(R33A)-miniCGBE and rA1(R33A)-CGBE, currently the most popular choices for C-to-G base editing [27, 29], were also included in the analysis (Fig. 4). While the rA1(R33A)- miniCGBE/CGBE demonstrated very low editing efficiency, averaging around 3% and 2%, respectively, across all tested sites, and displayed only slightly better performance in TC contexts, as previously reported [27, 31], the CDA1 CGBEs exhibited better compatibility with various substrate sequence contexts and displayed significantly higher efficiency of C-to-G editing at most of the tested sites. In particular with the truncated version CDA1Δ194-CGBE, which displayed the best performance at almost all tested sites, an average editing efficiency of 22% was achieved, representing an up to 11-fold increase compared to the reference editor rA1(R33A)-CGBE (Fig. 4). We also compared CDA1Δ195-CGBE and CDA1Δ194-CGBE across the 15 target sites. The results showed similar editing efficiencies, with CDA1Δ194-CGBE exhibiting slightly higher editing levels than CDA1Δ195-CGBE (on average 23.44% versus 20.16%; Supplementary Fig. S3). To more comprehensively investigate the motif preference of CDA1 CGBEs, we tested the efficiency of the CDA1Δ194-CGBE on an additional 21 target sites covering all NCN motif contexts (Supplementary Fig. S4). The results obtained indicate that, despite observing low editing efficiency in a limited number of NCNs (ACT, ACG, and CCT), the CDA1Δ194-CGBE was compatible with the majority of sequence contexts, exhibiting efficiencies ranging from 10% to 53.7% (Supplementary Fig. S4). These results highlight the potential of CDA1-derived CGBEs, especially in combination with the optimal CDA1 truncation, as a promising choice for various editing scenarios and as novel tools that greatly expand the applicability of C-to-G base editing.

In an attempt to further enhance the efficiency of C-to-G editing, we introduced rXRCC1, a BER protein known to increase C-to-G editing efficiency upon fusion with the rAPOBEC-nCas9 complex [27]. We applied this fusion strategy to both CDA1Δ194-miniCGBE and CDA1Δ194-CGBE, generating CDA1Δ194-XRCC1-miniCGBE and CDA1Δ194-XRCC1-CGBE (Supplementary Fig. S5A). Subsequently, we analyzed the editing profiles of these fusions at three target sites. However, we observed no significant increase in C-to-G editing efficiency at any of the tested sites for most of the fusions, with the exception of CDA1Δ194-XRCC1-CGBE at the polyC-2 site, where a moderate (nearly two-fold) increase was observed (Supplementary Fig. S5B).

Since fusing CDA1 to the C-terminus of nCas9 has been shown to improve editing efficiency compared to fusion to the N-terminus (Fig. 1), we tested whether this modification also enhances editing efficiencies by fusing the truncated CDA1 to the C-terminus of nCas9. To this end, we generated cCDA1Δ194-CGBE and included the N-terminally fused CDA1Δ194-CGBE and the full-length CDA1-harboring C-terminal fusion cCDA1-CGBE for comparison. Editing efficiencies were assessed for 15 target sites (Supplementary Fig. S6). The results showed that the truncated CDA1 fused to the C-terminus of nCas9 exhibited similar editing efficiency as the full-length CDA1 fusion, but both were less efficient than CDA1Δ194-CGBE. This may be due to the fact that truncating CDA1 and fusing it to the C-terminus of Cas9 did not significantly alter its editing precision or efficiency, as previously reported by us [26].

Recently, two glycosylase-based editors, DAF-CBE and CE-CDG, have been developed that facilitate C-to-G editing [43, 44]. To evaluate the performance of our CDA1Δ194-CGBE in comparison with these recently published variants, we constructed vectors for both DAF-CBE and CE-CDG and conducted editing experiments across 10 target sites. The comparative analysis demonstrated that CDA1Δ194-CGBE achieved the highest average editing efficiency at position C_3_, with values of 22.57%, 15.04%, and 10.76% for CDA1Δ194-CGBE, DAF-CBE, and CE-CDG, respectively (Supplementary Fig. S7). These results indicate an enhancement in editing efficiency provided by CDA1Δ194-CGBE when targeting the C_3_ position compared to the other two glycosylase-based editors.

### Analysis of genome-wide off-target DNA editing for CDA1-based CGBEs

CBEs were reported to produce substantial genome-wide off-target effects that are largely independent of the sgRNA [45, 46] and rather caused by the property of deaminases [47] to possess an intrinsic DNA-binding affinity, thus triggering non-specific deamination. In the case of CDA1, potential DNA-binding regions were identified between residues 21-27 and 172-192 of the (208 amino acid long) protein [48]. Our prior work had demonstrated that even C-terminal truncations making CDA1 as short as 158 amino acids still largely retain the CBE activity [26]. Moreover, truncated CDA1 versions were found to significantly reduce off-target editing [26, 48].

**Figure 5.**
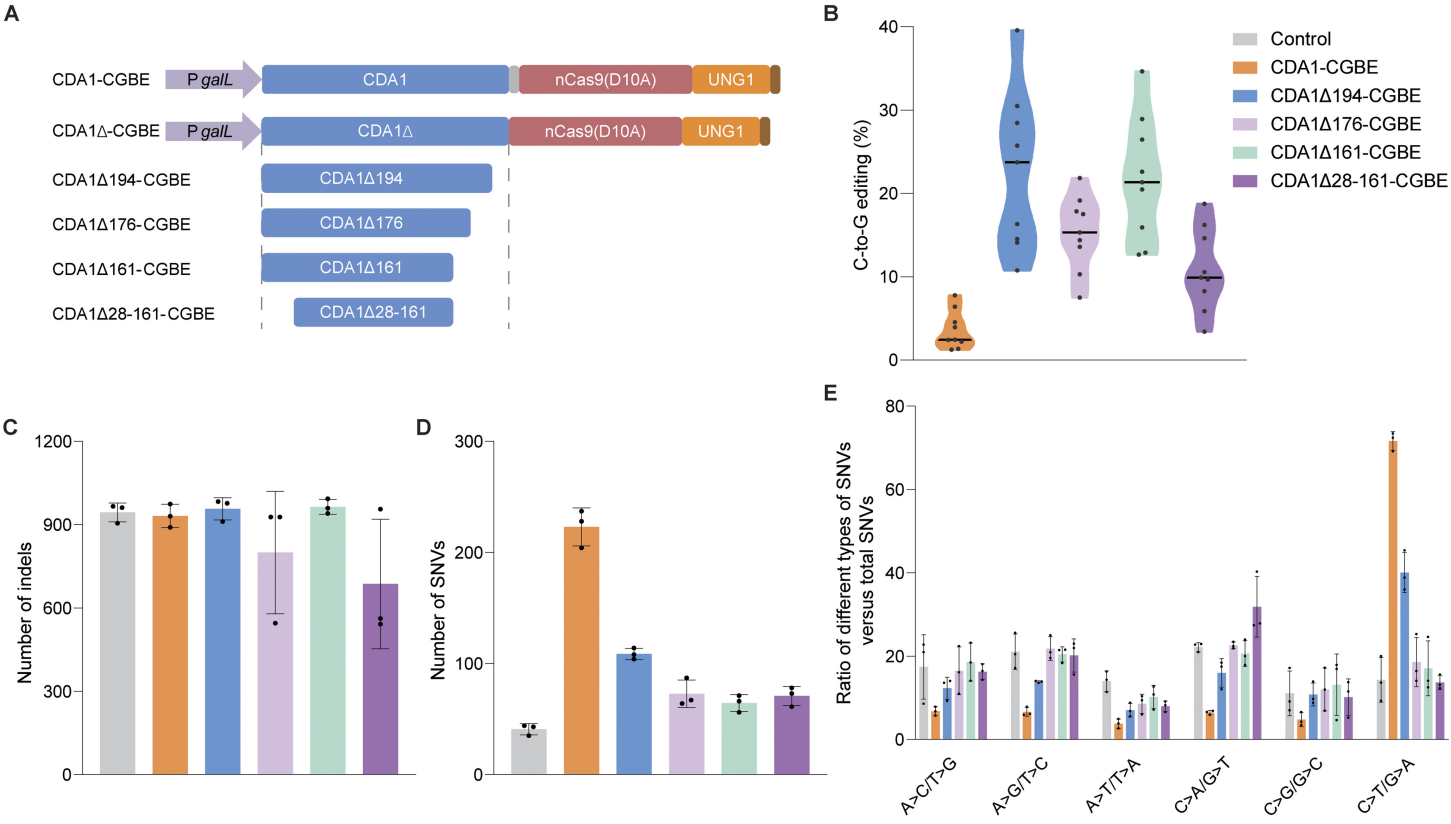
Analysis of off-target editing. Whole-genome sequencing was performed to identify on-target and off-target mutations in strains expressing CDA1 CGBE, CDA1Δ194-CGBE, CDA1Δ176-CGBE, CDA1Δ161-CGBE, and CDA1Δ28-161-CGBE, or harboring a control plasmid lacking a BE construct. (**A**) Schematic representation of the architecture of the constructed CGBEs featuring a series of truncated CDA1 variants. (**B**) Efficiency assessment of C-to-G base editing for the four CGBEs. Comparison of the total number of detected indels (**C**) and SNVs (**D**) by the BEs. (**E**) Mutation frequencies of different types of SNVs (single-nucleotide variants) in cells treated with the five BEs and the control strain. The sgRNA used was designed to target site *Can1-5* (Supplementary Table S1). Values and error bars represent the mean and standard deviation of three independent biological replicates. Source data are provided as a Source Data file in the Supplementary data.

In an effort to minimize the frequency of off-target editing, we generated a series of eight CGBEs with various C- and/or N-terminal truncations of CDA1 (Fig. 5A). To evaluate the impact of these truncations on C-to-G editing efficiency, we analyzed editing in the three polyC sites (Fig. 5B and Supplementary Fig. S8). The results showed that further CDA1 truncations (beyond amino acid residue 188) reduced C-to-G editing efficiency to varying degrees, compared to the best-performing version CDA1Δ194-CGBE. This reduction was particularly pronounced for the version with truncations at both termini, CDA1Δ28-161-CGBE, which displayed a 50% decrease in efficiency. By contrast, the C-terminally truncated variant CDA1Δ161-CGBE maintained a remarkable editing efficiency of up to 90% (Fig. 5B and Supplementary Fig. S8).

We next investigated off-target editing in yeast cells treated with CDA1 CGBE, four truncated CDA1 versions (CDA1Δ194-CGBE, CDA1Δ176-CGBE, CDA1Δ161-CGBE, and CDA1Δ28-161-CGBE), and a no BE control, in combination with an sgRNA targeting the *Can1*-5 site (Fig. 5C–E). Canavanine selection was used to isolate colonies harboring on-target editing events, as previously described [25]. For all constructs, cultures grown from three different transformed colonies were mixed, followed by genomic DNA isolation and whole-genome sequencing, as previously described [25]. As expected, all five CGBEs showed comparable numbers of indels as the no BE control (Fig. 5C). However, when analyzing the total number of SNVs, the full-length fusions demonstrated a notably higher count of SNVs compared to the control, consistent with previous reports on off-target effects of cytosine BEs [25, 45, 46]. By contrast, the four truncated versions displayed a significantly reduced number of SNVs that was only slightly higher than that of the negative control (Fig. 5C). Examination of the mutation types revealed that the CDA1 CGBE exhibited a notably higher frequency of C-to-T (G-to-A) transitions compared to the control and the truncated BEs CDA1Δ-CGBEs (Fig. 5E). Importantly, the three versions carrying larger C-terminal deletions, CDA1Δ176-CGBE, CDA1Δ161-CGBE, and CDA1Δ28-161-CGBE, showed a marked reduction in off-target DNA editing, reaching levels that were comparably low as in the control. We also conducted a comparative analysis of the genome-wide DNA off-target activity of CDA1Δ194-CGBE, the commonly used rA1(R33A)-CGBE, and eA3A-CGBE. The results showed that these three BEs exhibited comparable off-target editing levels that were only slightly higher than that of the control. These data are consistent with previous reports on off-target editing in their respective CBE derivatives (Supplementary Fig. S9) [25, 49]. Moreover, they indicate that high editing precision of BEs can contribute to reduced non-specific editing at off-target sites.

To also explore whether our new CDA1 CGBEs cause RNA off-target editing due to their cytidine deaminase activity, we evaluated the number of RNA C-to-U SNVs in strains treated with our two best-performing CGBE variants (CDA1Δ194-CGBE and CDA1Δ161-CGBE). For comparison, we also included the eA3A-CGBE, the rA1(R33A)-CGBE, whose CBE derivatives had been previously shown to significantly reduce RNA off-target edits, and a control strain. The results revealed that both CDA1Δ-CGBEs induced RNA edits at levels that were comparably low as those in the negative control. Furthermore, they exhibited either comparable or even lower levels of off-target C-to-U RNA editing compared to the eA3A-CGBE and the rA1(R33A)-CGBE (Supplementary Fig. S10), consistent with a previous report [50].

### Enhanced C-to-G editing with truncated CDA1 variants in human and plant cells

**Figure 6.**
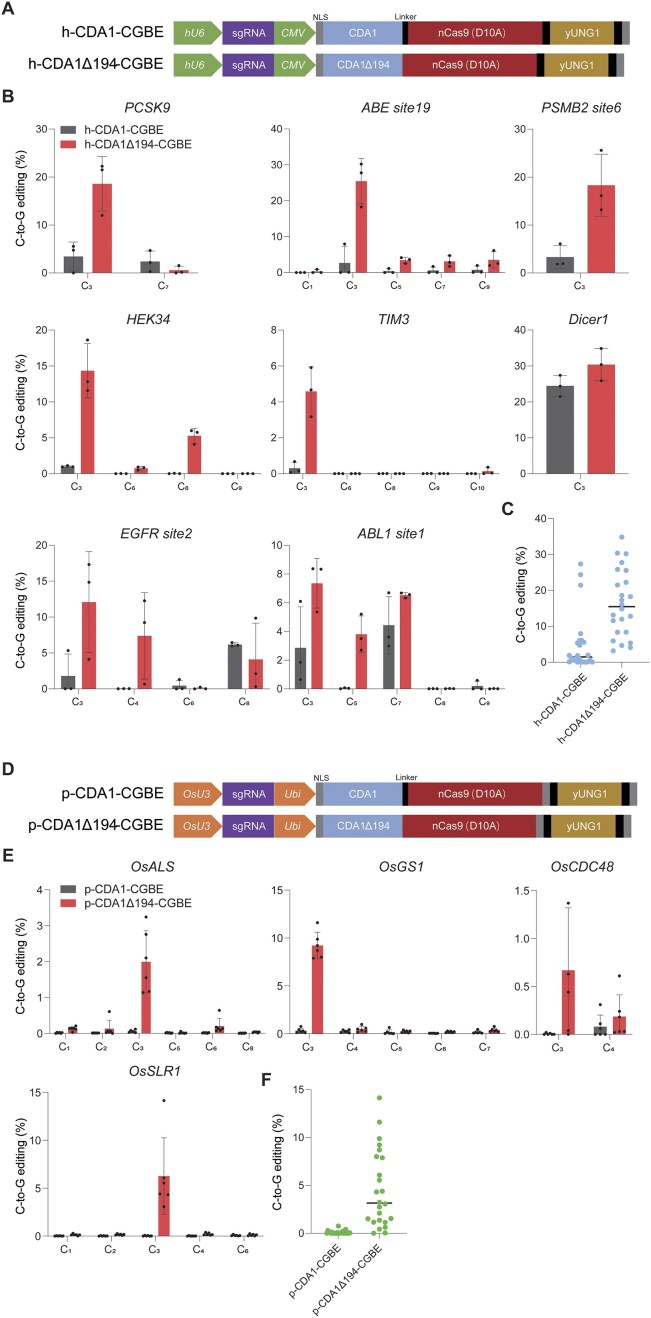
Improvement of C-to-G editing efficiency with truncated CDA1 variants in human and plant cells. (**A**) Schematic map of the structures of the BE constructs h-CDA1-CGBE and h-CDA1Δ194-CGBE for human cells. See the “Materials and methods” section for details. (**B**) Comparison of C-to-G editing frequencies of h-CDA1-CGBE and h-CDA1Δ194-CGBE at eight endogenous sites in human cells. The number below each target C on the *x*-axis indicates its distance from the distal end of the PAM. Values and error bars represent the mean and standard deviation of three biological replicates. (**C**) Summary of C-to-G editing frequencies induced by the two CGBEs at eight target sites, with the horizontal lines representing the median values. Each dot represents the editing frequency in an individual biological replicate. (**D**) Schematic maps of the p-CDA1-CGBE and p-CDA1Δ194-CGBE constructs for rice cells. (**E**) Comparison of C-to-G editing frequencies of p-CDA1-CGBE and p-CDA1Δ194-CGBE at four endogenous sites in the rice genome. The number below each target C on the *x*-axis indicates its distance from the distal end of the PAM. Values and error bars represent the mean and standard deviation of six biological replicates. (**F**) Summary of C-to-G editing frequencies induced by the two CGBEs at four target sites, with the horizontal lines representing the median value. Each dot represents the editing frequency in an individual biological replicate. Source data for panels (B), (C), (E), and (F) are provided as a Source Data file in the Supplementary data.

To further investigate the potential of our novel CDA1Δ-CGBEs in enhancing C-to-G editing efficiency in higher eukaryotic cells, we assessed the editing performance of the best-performing CGBE variant, CDA1Δ194-CGBE, for human cells (referred to as variant h-CDA1Δ194-CGBE) and rice cells (variant p-CDA1Δ194-CGBE) and compared the editing profiles to those of the corresponding full-length CDA1 BEs (h-CDA1-CGBE and p-CDA1-CGBE, respectively) as controls (Fig. 6). C-to-G editing was evaluated at eight endogenous target sites in the human genome and four endogenous target sites in the rice genome. The results showed that, similar to our observations in yeast, C-to-G editing predominantly occurred at position C_3_ across all target sites in both human and rice cells. Remarkably, CDA1Δ194-CGBE treatment also resulted in significantly higher editing frequencies compared to the full-length BEs. In human cells, editing frequencies were increased by 1.24-15.01-fold with CDA1Δ194-CGBE, yielding an average editing frequency of ∼16.4%, compared to only 5% with the full-length control (Fig. 6B and C). In rice cells, the improvement was even more pronounced, with editing frequencies increasing 27.11-215.07-fold, reaching an average of 4.1% with CDA1Δ194-CGBE, compared to just 0.1% with the full-length BE (Fig. 6E and F)). These findings indicate that using truncated CDA1 variants to enhance C-to-G editing efficiency is effective not only in yeast but also in animal and plant cells.

## Discussion

In this study, we have described the development of CDA1-based high-precision CGBEs that enable efficient C-to-G base editing, predominantly at position 3 (relative to the distal end of PAM), a region that was previously inaccessible to CGBEs. We also demonstrated that, compared to APOBEC-based CGBEs, our new CGBEs exhibit better compatibility with a wide range of substrate sequence contexts. We have identified optimized CDA1 truncations that effectively minimize off-target DNA editing by CGBEs throughout the genome. Thus, our new CGBEs enrich the existing suite of base editing reagents and contribute to providing researchers with a growing arsenal of tools to precisely modify the genetic information and unlock new possibilities for gene therapy and molecular breeding.

The CGBEs constructed by fusing CDA1 to the C-terminus of nCas9 exhibited a higher efficacy in C-to-G editing than the fusion of CDA1 to the N-terminus of nCas9 (Fig. 1). We had previously shown that CDA1-derived CBEs exhibited different activity windows depending on the orientation of the fusion, in that the C-terminal fusion of nCas9 (known as cCDA1-BE3 [26] or Target-AID [21]) resulted in a relatively narrow activity window (C_2_-C_5_), while the N-terminal fusion (referred to as nCDA1-BE3 [26]) displayed a much wider editing window (C_1_-C_7_). A similar correlation between activity window and editing efficiently was also observed for other deaminases such as APOBEC1, in that variants with narrower windows (R33A [41] or YE1 [51]) showed significantly enhanced editing efficiency, including C-to-G editing efficiency when applied to CGBEs [29, 31]. Together, these findings strongly suggest that deaminases with narrower activity windows can increase the C-to-G efficiency of base editing, although the underlying mechanism requires further investigation. Consequently, the test of different deaminases or engineered variants thereof can potentially further diversify the existing CGBE toolkit.

Our systematic analysis of C-terminally truncated CDA1 variants resulted in increased efficiency of C-to-G editing, with the level of enhancement varying depending on the extent of the truncation. Comparison of all truncated versions of CDA1 revealed that CDA1Δ194-CGBE outperforms the others in terms of efficiency and accuracy. Therefore, CDA1Δ194-CGBE is strongly recommended for most applications. It is worth noting that CDA1Δ194-CGBE still generates some off-target editing in the genome, although to a much lesser extent than the full-length version. Additionally, we found that a larger CDA1 truncation, CDA1Δ161-CGBE, exhibited even higher C-to-G editing specificity (i.e. fewer off-target edits) with only mildly decreased editing efficiency (Fig. 5 and Supplementary Fig. S8). CGBEs carrying this larger truncation can be considered for scenarios where specificity is of utmost importance.

Despite having achieved remarkably high C-to-G editing efficiency through the use of optimized CDA1 truncations, the levels of C-to-G base editing remain lower than those obtainable upon C-to-T base editing using the corresponding CBEs. As this may pose a limitation to certain applications such as *in vivo* gene therapy, future efforts should be directed toward further increasing C-to-G editing efficiency. Recent advancements include the identification of DNA repair components that impact C-to-G editing efficiency through CRISPR interference screens, as well as the fusion of these components with deaminases and Cas proteins to enhance C-to-G editing activities [28]. Furthermore, the exploitation of engineered deaminases has also been reported to improve C-to-G editing efficiency [31]. These insights provide a solid foundation for the future development of new generations of CGBEs with further enhanced C-to-G editing efficiency.

It is noteworthy that some sites still show relatively low C-to-G editing efficiency, although sharing the local sequence context with efficiently edited sites. It is well known that the efficiency of base editing depends on a number of factors [52]. In addition to the local sequence context surrounding the target nucleotide, which determines sequence-dependent deaminase activity, sgRNA choice also plays a crucial role in base editing efficiency, by governing target DNA recognition and binding [52, 53]. Optimization of gRNA design can substantially enhance editing efficiency and improve target specificity [54]. Thus, selection of a successful target for efficient C-to-G base editing requires consideration of both the local sequence context and the general sgRNA design principles, as reported previously [55].

Importantly, our engineered CGBEs exhibited superior editing precision and compatibility with a wide range of substrate contexts. A narrower editing window implies fewer targeted nucleotides. To expand the scope of genome targeting, engineered Cas9 variants with modified PAM recognition properties (e.g. SpCas9-NG, SpG, and SpRY) and naturally occurring Cas9 orthologs (e.g. *Staphylococcus aureus* Cas9 and Cpf1 with different PAM specificities) can be used in conjunction with our BEs, although specific optimization may be necessary to account for differences in their three-dimensional structures.

In this work, we initially utilized diploid yeast, a eukaryotic model organism, to engineer a high-precision CGBE with a narrow editing window. Although it seemed reasonable to hypothesize that the new BE would be applicable also to higher eukaryotes, it was important to verify this assumption experimentally. We, therefore, confirmed the efficacy of our CGBE in both human and rice cells. Our results showed that, compared with the full-length CDA1, truncated CDA1-based CGBEs (CDA1Δ194-CGBEs) significantly enhance C-to-G editing efficiency and precision in both the plant and the animal system, suggesting that the differences in genome organization and chromatin structure between yeast, plant, and animal cells have only a limited impact on BE performance. These findings indicate that BEs identified as superior in one organism likely also display superior performance in other organisms, a conclusion that is consistent with a number of previous studies showing that most genome editing tools are readily transferrable between organisms while retaining their general properties and activity [21, 22, 25, 48]. This high level of adaptability is likely attributable to the simple molecular structure of CRISPR-Cas systems and the absence of species-specific factors influencing the editing reaction and/or target site recognition [2, 56].

Highly precise and efficient BEs will be crucial for future applications in genome editing, such as gene therapy, *in vivo* site-directed mutagenesis, and precision breeding in agriculture. Especially in gene therapy, where the correction of disease-causing mutations requires utmost precision without the risk of introducing unwanted mutations in the vicinity of the target nucleotide position (or elsewhere in the genome), our high-precision CGBEs are expected to find wide applications in basic and applied research.

## Supplementary Material

gkaf717_Supplemental_Files

## Data Availability

The data supporting the findings of this study are available within the paper and its supplementary information files. High-throughput sequencing data and Python scripts utilized in this research are accessible on Zenodo (10.5281/zenodo.15737495). The source data underlying Figs 1-6 and Supplementary Figs 1-10 are provided as a Source Data file in the supplementary data.

## Supplementary data

Supplementary data is available at NAR online.
