# Supplementary material for: Engineering of high-precision C-to-G base editors with expanded site selectivity and target compatibility
Source: Nucleic Acids Res. 2025 Aug 11;53(15):gkaf717. doi: 10.1093/nar/gkaf717 (PMC12342917; doi:10.1093/nar/gkaf717)
Supplement: gkaf717_Supplemental_Files [file gkaf717_supplemental_files.zip › Supplementary File-Corrected version.docx]

**Supplementary Information**

**Table S1.** **Target protospacer sequences analyzed in this study.**

Target Cs are shown in red, with the subscript numbers indicating their position relative to the distal end of the PAM. The PAM sequences are shown in blue.

| **Target** | **Sequence (5’ → 3’)** | **Analysis method** |
| --- | --- | --- |
| PolyC-1 | C_1_C_2_C_3_C_4_C_5_C_6_C_7_CATGTTCCGAGATCGG | NGS |
| PolyC-2 | AC_2_C_3_C_4_C_5_C_6_C_7_C_8_TAATATATTCAAAGG | NGS |
| PolyC-3 | GC_2_C_3_C_4_C_5_C_6_C_7_C_8_TCCCCAAAAAAATGG | NGS |
| *CAN1*-1 | TC_2_C_3_AATAACGGAATCCAACTGGG | NGS |
| *CAN1*-2 | GC_2_C_3_C_4_TGGAACTTAGTGTAGTTGG | NGS |
| *CAN1*-3 | TTC_3_TC_5_TATGGAGGATGGCATAGG | NGS |
| *CAN1*-4 | TGC_3_C_4_TCAATGTCTCTTCTATCGG | NGS |
| *CAN1*-5 | TAC_3_ATGGAGACATCTACTGGTGG | canavanine selection, NGS |
| *CAN1*-6 | GCTTAC_6_ATGGAGACATCTACTGG | canavanine selection |
| CGBE site 1 | ATC_3_CAC_6_TGTATACTCGTTACGGG | NGS |
| CGBE site 2 | CCC_3_CTC_6_AGGCTATGGACGTGCGG | NGS |
| CGBE site 3 | ATC_3_CTC_6_TAAACTCTTCTTGCTGG | NGS |
| CGBE site 4 | CTC_3_CTC_6_TGGTGTTAAAGGTATGG | NGS |
| CGBE site 5 | GTC_3_CTC_6_TATTACAACGGATCTGG | NGS |
| CGBE site 6 | GCC_3_CAC_6_TAATATGGTCCAGATGG | NGS |
| CGBE site 7 | ATC_3_CAC_6_CATTAAGACAAATTGGG | NGS |
| CGBE site 8 | AAC_3_CAC_6_CAGTAAGAAACTCTTGG | NGS |
| CGBE site 9 | TCC_3_CGC_6_TTGTACATTCATTCTGG | NGS |
| CGBE site 10 | TGC_3_GGC_6_ACTCTGAACATCGCTGG | NGS |
| CGBE site 11 | AGC_3_TTC_6_TTGCTGTAGGTTATGGG | NGS |
| CGBE site 12 | AGC_3_AGC_6_GGTGGGTTCTTATAAGG | NGS |
| CGBE site 13 | AGC_3_TTC_6_AACGTCGTGAAAGAGGG | NGS |
| CGBE site 14 | AGC_3_ATC_6_TTCGGAAACGATGAAGG | NGS |
| CGBE site 15 | TGC_3_TCC_6_TCTTTTCTCCTTTATGG | NGS |
| CGBE site 16 | TAC_3_GAATCGTCAATCGCTTGCGG | NGS |
| CGBE site 17 | AAC_3_CATACTGAGTCTGAAATTGG | NGS |
| CGBE site 18 | CAC_3_TAATTTACCGCCATTTCTGG | NGS |
| CGBE site 19 | AGC_3_GTAGTTAACCCCTCTGCAGG | NGS |
| CGBE site 20 | TGC_3_CATATCTATTGGGTATAAGG | NGS |
| CGBE site 21 | CGC_3_TTGTTAACTCTTTTCGTCGG | NGS |
| CGBE site 22 | TCC_3_AAACCCACATACAAACGCGG | NGS |
| CGBE site 23 | ACC_3_GTCCTTTTGAATTCGAATGG | NGS |
| CGBE site 24 | ACC_3_CTGGAATTGCAATAGCTTGG | NGS |
| CGBE site 25 | TCC_3_TTGGCCATTTCTTCTGCTGG | NGS |
| CGBE site 26 | ATC_3_AAGCTGGAATTTATTGGTGG | NGS |
| CGBE site 27 | CTC_3_GAATGTATACCCAGGAAAGG | NGS |
| CGBE site 28 | TTC_3_TTCAAGCTATGGAGGCCCGG | NGS |
| CGBE site 29 | AAC_3_ATGCCCTAAAGGCACCTCGG | NGS |
| CGBE site 30 | TAC_3_GAAGTCATTTAATTAAATGG | NGS |
| CGBE site 31 | GAC_3_CTTCGAAGGGAACGACTCGG | NGS |
| CGBE site 32 | GAC_3_TGTCATCCTATATAGGGTGG | NGS |
| CGBE site 33 | TTC_3_GCATCTGGATGCTATTATGG | NGS |
| CGBE site 34 | CTC_3_CAAACAGTGAAACTGCTAGG | NGS |
| CGBE site 35 | GTC_3_TTGTACTTAAAGGGCAGTGG | NGS |
| CGBE site 36 | TGC_3_TATAGGAGTCTGGTTACTGG | NGS |
| *ABL1* site1 | TGC_3_ACTCCCTCAGGTAGTCCAGG | NGS |
| *ABE* site19 | CAC_3_ACACACTTAGAATCTGTGGG | NGS |
| *Dicer1* | AAC_3_TTTATTGGAGATTTACTTGG | NGS |
| *EGFR* site2 | GTC_3_CACGCTGGCCATCACGTAGG | NGS |
| *HEK34* | TGC_3_TTCTCCAGCCCTGGCCTGGG | NGS |
| *PCSK9* | TGC_3_TTACCTGTCTGTGGAAGCGG | NGS |
| *PSMB2* site6 | TAC_3_AGGCGAAACAGGCAGACAGG | NGS |
| *TIM3* | TTC_3_TACACCCCAGCCGCCCCAGG | NGS |
| *OsALS* | CCC_3_ACCGCAATATGCCATTCAGG | NGS |
| *OsCDC48* | AAC_3_CACATGGTAAGCAGCTCAGG | NGS |
| *OsGS1* | GGC_3_CCCCGGCGAGGACAGTGAGG | NGS |
| *OsSLR1* | CCC_3_CTCGGACCTCTCCTCCTGGG | NGS |

**Table S2.** **Primers for construction of sgRNA-expressing plasmids.**

The protospacer sequences are shown in red.

| **Primer name** | **Sequence (5’ → 3’)** |
| --- | --- |
| gRNA-PolyC-1 | AAAGATAAATGATCGCCCCCCCCATGTTCCGAGATGTTTTAGAGCTAGAAATAGCAAGT |
| gRNA-PolyC-2 | AAAGATAAATGATCGACCCCCCCTAATATATTCAAGTTTTAGAGCTAGAAATAGCAAGT |
| gRNA-PolyC-3 | AAAGATAAATGATCGGCCCCCCCTCCCCAAAAAAAGTTTTAGAGCTAGAAATAGCAAGT |
| gRNA-*CAN1*-1 | AAAGATAAATGATCGTCCAATAACGGAATCCAACTGTTTTAGAGCTAGAAATAGCAAGT |
| gRNA-*CAN1*-2 | AAAGATAAATGATCGGCCCTGGAACTTAGTGTAGTGTTTTAGAGCTAGAAATAGCAAGT |
| gRNA-*CAN1*-3 | AAAGATAAATGATCGTTCTCTATGGAGGATGGCATGTTTTAGAGCTAGAAATAGCAAGT |
| gRNA-*CAN1*-4 | AAAGATAAATGATCGTGCCTCAATGTCTCTTCTATGTTTTAGAGCTAGAAATAGCAAGT |
| gRNA-*CAN1*-5 | AAAGATAAATGATCGTACATGGAGACATCTACTGGGTTTTAGAGCTAGAAATAGCAAGT |
| gRNA-*CAN1*-6 | AAAGATAAATGATCGGCTTACATGGAGACATCTACGTTTTAGAGCTAGAAATAGCAAGT |
| gRNA-CGBE-1 | AAAGATAAATGATCGATCCACTGTATACTCGTTACGTTTTAGAGCTAGAAATAGCAAGT |
| gRNA-CGBE-2 | AAAGATAAATGATCGCCCCTCAGGCTATGGACGTGGTTTTAGAGCTAGAAATAGCAAGT |
| gRNA-CGBE-3 | AAAGATAAATGATCGATCCTCTAAACTCTTCTTGCGTTTTAGAGCTAGAAATAGCAAGT |
| gRNA-CGBE-4 | AAAGATAAATGATCGCTCCTCTGGTGTTAAAGGTAGTTTTAGAGCTAGAAATAGCAAGT |
| gRNA-CGBE-5 | AAAGATAAATGATCGGTCCTCTATTACAACGGATCGTTTTAGAGCTAGAAATAGCAAGT |
| gRNA-CGBE-6 | AAAGATAAATGATCGGCCCACTAATATGGTCCAGAGTTTTAGAGCTAGAAATAGCAAGT |
| gRNA-CGBE-7 | AAAGATAAATGATCGATCCACCATTAAGACAAATTGTTTTAGAGCTAGAAATAGCAAGT |
| gRNA-CGBE-8 | AAAGATAAATGATCGAACCACCAGTAAGAAACTCTGTTTTAGAGCTAGAAATAGCAAGT |
| gRNA-CGBE-9 | AAAGATAAATGATCGTCCCGCTTGTACATTCATTCGTTTTAGAGCTAGAAATAGCAAGT |
| gRNA-CGBE-10 | AAAGATAAATGATCGTGCGGCACTCTGAACATCGCGTTTTAGAGCTAGAAATAGCAAGT |
| gRNA-CGBE-11 | AAAGATAAATGATCGAGCTTCTTGCTGTAGGTTATGTTTTAGAGCTAGAAATAGCAAGT |
| gRNA-CGBE-12 | AAAGATAAATGATCGAGCAGCGGTGGGTTCTTATAGTTTTAGAGCTAGAAATAGCAAGT |
| gRNA-CGBE-13 | AAAGATAAATGATCGAGCTTCAACGTCGTGAAAGAGTTTTAGAGCTAGAAATAGCAAGT |
| gRNA-CGBE-14 | AAAGATAAATGATCGAGCATCTTCGGAAACGATGAGTTTTAGAGCTAGAAATAGCAAGT |
| gRNA-CGBE-15 | AAAGATAAATGATCGTGCTCCTCTTTTCTCCTTTAGTTTTAGAGCTAGAAATAGCAAGT |
| gRNA-CGBE-16 | AAAGATAAATGATCGTACGAATCGTCAATCGCTTGGTTTTAGAGCTAGAAATAGCAAGT |
| gRNA-CGBE-17 | AAAGATAAATGATCGAACCATACTGAGTCTGAAATGTTTTAGAGCTAGAAATAGCAAGT |
| gRNA-CGBE-18 | AAAGATAAATGATCGCACTAATTTACCGCCATTTCGTTTTAGAGCTAGAAATAGCAAGT |
| gRNA-CGBE-19 | AAAGATAAATGATCGAGCGTAGTTAACCCCTCTGCGTTTTAGAGCTAGAAATAGCAAGT |
| gRNA-CGBE-20 | AAAGATAAATGATCGTGCCATATCTATTGGGTATAGTTTTAGAGCTAGAAATAGCAAGT |
| gRNA-CGBE-21 | AAAGATAAATGATCGCGCTTGTTAACTCTTTTCGTGTTTTAGAGCTAGAAATAGCAAGT |
| gRNA-CGBE-22 | AAAGATAAATGATCGTCCAAACCCACATACAAACGGTTTTAGAGCTAGAAATAGCAAGT |
| gRNA-CGBE-23 | AAAGATAAATGATCGACCGTCCTTTTGAATTCGAAGTTTTAGAGCTAGAAATAGCAAGT |
| gRNA-CGBE-24 | AAAGATAAATGATCGACCCTGGAATTGCAATAGCTGTTTTAGAGCTAGAAATAGCAAGT |
| gRNA-CGBE-25 | AAAGATAAATGATCGTCCTTGGCCATTTCTTCTGCGTTTTAGAGCTAGAAATAGCAAGT |
| gRNA-CGBE-26 | AAAGATAAATGATCGATCAAGCTGGAATTTATTGGGTTTTAGAGCTAGAAATAGCAAGT |
| gRNA-CGBE-27 | AAAGATAAATGATCGCTCGAATGTATACCCAGGAAGTTTTAGAGCTAGAAATAGCAAGT |
| gRNA-CGBE-28 | AAAGATAAATGATCGTTCTTCAAGCTATGGAGGCCGTTTTAGAGCTAGAAATAGCAAGT |
| gRNA-CGBE-29 | AAAGATAAATGATCGAACATGCCCTAAAGGCACCTGTTTTAGAGCTAGAAATAGCAAGT |
| gRNA-CGBE-30 | AAAGATAAATGATCGTACGAAGTCATTTAATTAAAGTTTTAGAGCTAGAAATAGCAAGT |
| gRNA-CGBE-31 | AAAGATAAATGATCGGACCTTCGAAGGGAACGACTGTTTTAGAGCTAGAAATAGCAAGT |
| gRNA-CGBE-32 | AAAGATAAATGATCGGACTGTCATCCTATATAGGGGTTTTAGAGCTAGAAATAGCAAGT |
| gRNA-CGBE-33 | AAAGATAAATGATCGTTCGCATCTGGATGCTATTAGTTTTAGAGCTAGAAATAGCAAGT |
| gRNA-CGBE-34 | AAAGATAAATGATCGCTCCAAACAGTGAAACTGCTGTTTTAGAGCTAGAAATAGCAAGT |
| gRNA-CGBE-35 | AAAGATAAATGATCGGTCTTGTACTTAAAGGGCAGGTTTTAGAGCTAGAAATAGCAAGT |
| gRNA-CGBE-36 | AAAGATAAATGATCGTGCTATAGGAGTCTGGTTACGTTTTAGAGCTAGAAATAGCAAGT |
| sgRNA-Rev | TATAGGGCGAATTGGGTACCGGCCGCAAATTAAAG |
| gRNA-*ABL1* site1 | AAACTGCACTCCCTCAGGTAGTCCTGTT |
| gRNA-*ABE* site19 | AAACCACACACACTTAGAATCTGTTGTT |
| gRNA-*Dicer1* | AAACAACTTTATTGGAGATTTACTTGTT |
| gRNA-*EGFR* site2 | AAACGTCCACGCTGGCCATCACGTTGTT |
| gRNA-*HEK34* | AAACTGCTTCTCCAGCCCTGGCCTTGTT |
| gRNA-*PCSK9* | AAACTGCTTACCTGTCTGTGGAAGTGTT |
| gRNA-*PSMB2* site6 | AAACTACAGGCGAAACAGGCAGACTGTT |
| gRNA-*TIM3* | AAACTTCTACACCCCAGCCGCCCCTGTT |
| gRNA-*OsALS* | GGCGCCCACCGCAATATGCCATTCGTTT |
| gRNA-*OsCDC48* | GGCGAACCACATGGTAAGCAGCTCGTTT |
| gRNA-*OsGS1* | GGCGGGCCCCCGGCGAGGACAGTGGTTT |
| gRNA-*OsSLR1* | GGCGCCCCTCGGACCTCTCCTCCTGTTT |

**Table S3.** **Primers for construction of base editors.**

| **Primer name** | **Sequence (5’ → 3’)** | **Constructs** |
| --- | --- | --- |
| nBE-F | GGATTCTAGAACTAGTGGATCC | A1/A1(R33A)/A3A/eA3A/nCDA1-nCas9 |
| nBE-R | TTACTAAAGATCTCCTGCAGG |  |
| cCDA1-miniCGBE-1F | ACCAACTTGGGCGCGCCTGC | cCDA1-miniCGBE |
| cCDA1-miniCGBE-1R | CCACCAGATCTAGAAACAGCAGG |  |
| cCDA1-miniCGBE-2F | TTCTAGATCTGGTGGTTCACCCA |  |
| cCDA1-miniCGBE-2R | CATTTGCTCGGCATGCCGGTAG |  |
| cCDA1-CGBE-1F | ACCAACTTGGGCGCGCCTGC | cCDA1-CGBE |
| cCDA1-CGBE-1R | CACCACATTGAACCACCAGATCT |  |
| cCDA1-CGBE-2F | TGGTTCAATGTGGTGCATGAGAAG |  |
| cCDA1-CGBE-2R | CACCAGAAGGGTCCTTTGATTCT |  |
| cCDA1-CGBE-3F | AGGACCCTTCTGGTGGTTCACCCAAG |  |
| cCDA1-CGBE-3R | CATTTGCTCGGCATGCCGGTAG |  |
| nBE-miniCGBE-1F | ACCAACTTGGGCGCGCCTGC | CDA1-miniCGBE |
| nBE-miniCGBE-1R | GGGTGAACCACCAGAGTCTCCAC |  |
| nBE-miniCGBE-2F | TCTGGTGGTTCACCCAAGAAGAAGAGGAAGG |  |
| nBE-miniCGBE-2R | ACATGCGTACACGCGTCTG |  |
| nBE-CGBE-1F | ACCAACTTGGGCGCGCCTGC | CDA1-CGBEs |
| nBE-CGBE-1R | CACCACATTGAACCACCAGAGTCTC |  |
| nBE-CGBE-2F | TGGTTCAATGTGGTGCATGAGAAG |  |
| nBE-CGBE-2R | CACCAGAAGGGTCCTTTGATTCT |  |
| nBE-CGBE-3F | AGGACCCTTCTGGTGGTTCACCCAAG |  |
| nBE-CGBE-3R | ACATGCGTACACGCGTCTG |  |
| CDA1Δ-1F | GGATTCTAGAACTAGTGGATCC | CDA1(Δ195-Δ150)-BEs |
| CDA1Δ-2R | TTACTAAAGATCTCCTGCAGG |  |
| CDA1(Δ195)-1R | TCTTGTCCATAATCATAATGGACAACTCGCTC | CDA1(Δ195)-BEs |
| CDA1(Δ195)-2F | CATTATGATTATGGACAAGAAGTACTCCAT |  |
| CDA1(Δ194)-1R | TCTTGTCCATCATAATGGACAACTCGCTC | CDA1(Δ194)-BEs |
| CDA1(Δ194)-2F | GTCCATTATGATGGACAAGAAGTACTCCAT |  |
| CDA1(Δ193)-1R | TCTTGTCCATAATGGACAACTCGCTCCGTCG | CDA1(Δ193)-BEs |
| CDA1(Δ193)-2F | GTTGTCCATTATGGACAAGAAGTACTCCAT |  |
| CDA1(Δ192)-1R | TCTTGTCCATGGACAACTCGCTCCGTCGTTTT | CDA1(Δ192)-BEs |
| CDA1(Δ192)-2F | CGAGTTGTCCATGGACAAGAAGTACTCCAT |  |
| CDA1(Δ190)-1R | TCTTGTCCATCTCGCTCCGTCGTTTTTCAGC | CDA1(Δ190)-BEs |
| CDA1(Δ190)-2F | ACGGAGCGAGATGGACAAGAAGTACTCCAT |  |
| CDA1(Δ188)-1R | TCTTGTCCATCCGTCGTTTTTCAGCTCGCT | CDA1(Δ188)-BEs |
| CDA1(Δ188)-2F | AAAACGACGGATGGACAAGAAGTACTCCAT |  |
| CDA1(Δ182)-1R | TCTTGTCCATCTTCAAAGTCTTCTCAAGCC | CDA1(Δ182)-BEs |
| CDA1(Δ182)-2F | GACTTTGAAGATGGACAAGAAGTACTCCAT |  |
| CDA1(Δ176)-1R | TCTTGTCCATCCATCTATTCTCATTCAATT | CDA1(Δ176)-BEs |
| CDA1(Δ176)-2F | GAATAGATGGATGGACAAGAAGTACTCCAT |  |
| CDA1(Δ167)-1R | TCTTGTCCATCGACGATTGGATGAATATTT | CDA1(Δ167)-BEs |
| CDA1(Δ167)-2F | CCAATCGTCGATGGACAAGAAGTACTCCAT |  |
| CDA1(Δ161)-1R | TCTTGTCCATTTTCCTGCAACATTGGTAGT | CDA1(Δ161)-BEs |
| CDA1(Δ161)-2F | TTGCAGGAAAATGGACAAGAAGTACTCCAT |  |
| CDA1(Δ158)-1R | TCTTGTCCATACATTGGTAGTGTTCACTTA | CDA1(Δ158)-BEs |
| CDA1(Δ158)-2F | CTACCAATGTATGGACAAGAAGTACTCCAT |  |
| CDA1(Δ150)-1R | TCTTGTCCATTACATTCAACCCAACC | CDA1(Δ150)-BEs |
| CDA1(Δ150)-2F | ATGTAATGGACAAGAAGTACTCCAT |  |
| CDA1(ΔN28)-F | GGATTCTAGAACTAGTGGATCCCCCGGGAAAAAAATGTCCGTGTCGCATAGATGCTACG | CDA1(ΔN28)-BEs |
| CDA1(ΔN28)-R | TTACTAAAGATCTCCTGCAGG |  |
| CDA1(ΔN28-161)-1F | GGATTCTAGAACTAGTGGATCCCCCGGGAAAAAAATGTCCGTGTCGCATAGATGCTACG | CDA1(ΔN28-161)-BEs |
| CDA1(ΔN28-161)-1R | TCTTGTCCATTTTCCTGCAACATTGGTAGT |  |
| CDA1(ΔN28-161)-2F | TTGCAGGAAAATGGACAAGAAGTACTCCAT |  |
| CDA1(ΔN28-161)-2R | TTACTAAAGATCTCCTGCAGG |  |
| miniCGBE-rXRCC1-1F | ACCAACTTGGGCGCGCCTGC | nCDA1(Δ194)- -rXRCC1-miniCGBE |
| miniCGBE-rXRCC1-1R | TGAACCACCAGAGTCTCCACCGAGCTG |  |
| miniCGBE-rXRCC1-2F | GACTCTGGTGGTTCAATGCCTGAGATAAGC |  |
| miniCGBE-rXRCC1-2R | GGGTGAACCACCAGATGCCTGGGGTA |  |
| miniCGBE-rXRCC1-3F | TCTGGTGGTTCACCCAAGAAGAAGAGG |  |
| miniCGBE-rXRCC1-3R | ACATGCGTACACGCGTCTG |  |
| CGBE-rXRCC1-1F | ACCAACTTGGGCGCGCCTGC | nCDA1(Δ194)- rXRCC1-CGBE |
| CGBE-rXRCC1-1R | TGAACCACCAGAAGGGTCCTTTGATTC |  |
| CGBE-rXRCC1-2F | CCTTCTGGTGGTTCAATGCCTGAGATAAGC |  |
| CGBE-rXRCC1-2R | ACATGCGTACACGCGTCTG |  |
| h-CDA1-F | TAGAGATCCGCGGCCGCTAA |  |
| h-CDA1-R | TTCTTGTCCATAGACTCAGGTGTGGCGC | h-CDA1-CGBE |
| h-CDA1(Δ194)-R | TTGTCCATCATGATGCTCAGTTCGCTT | h-CDA1(Δ194)-CGBE |
| p-CDA1-F | TGTTACTTCTGCAGCCCTAGG |  |
| p-CDA1-R | TTGGAGAAAATCTCCTGCAG | p-CDA1-CGBE |
| p-CDA1(Δ194)-R | GTACTTCTTGTCCATCATGATGGACAGCTCACTCC | p-CDA1(Δ194)-CGBE |
| cCDA1(Δ194)-R | ACCACCAGATCTAGACATAATGGACAACTCGCTCCGTC | cCDA1(Δ194)-CGBE |
| CDG-F | ATTATGAATTTTTTCAAGGGTTCTTCTGGTTCCATGTTCTTTTCTCCATCACC | CE-CDG |
| CDG-R | TTCTCCGTTTGTTTCGGAACCAGAAGAACCCAATTCCTTCCAGTCAATTG |  |
| CDG4-F | TGGATCCCCCGGGAAAAAAATGTTCGGTGAATCTTGGAAGAA | DAF-CBE |
| CDG4-R | AGTTTCAGAACCAGACAATTCAGTCCAGTCGATTG |  |

**Table S4.** Primers for fragment amplification for next-generation sequencing (NGS) of target sites.

| **Primer name** | **Sequence (5’ → 3’)** |
| --- | --- |
| PolyC-1-NGS-1-F | ATCACGACTGCGGAAGTGAGGGGAGC |
| PolyC-1-NGS-1-R | GCCTAATATCCGTGCGCGTAATCCTTCT |
| PolyC-1-NGS-2-F | CGATGTACTGCGGAAGTGAGGGGAGC |
| PolyC-1-NGS-2-R | TGGTCATATCCGTGCGCGTAATCCTTCT |
| PolyC-1-NGS-3-F | AGTTCCACTGCGGAAGTGAGGGGAGC |
| PolyC-1-NGS-3-R | CTCTACTATCCGTGCGCGTAATCCTTCT |
| PolyC-1-NGS-4-F | CACTCAACTGCGGAAGTGAGGGGAGC |
| PolyC-1-NGS-4-R | TGTTGGTATCCGTGCGCGTAATCCTTCT |
| PolyC-1-NGS-5-F | GTGGCCACTGCGGAAGTGAGGGGAGC |
| PolyC-1-NGS-5-R | CGAAACTATCCGTGCGCGTAATCCTTCT |
| PolyC-1-NGS-6-F | CGTACGACTGCGGAAGTGAGGGGAGC |
| PolyC-1-NGS-6-R | CCACTCTATCCGTGCGCGTAATCCTTCT |
| PolyC-1-NGS-7-F | GGTAGCACTGCGGAAGTGAGGGGAGC |
| PolyC-1-NGS-7-R | ATCAGTTATCCGTGCGCGTAATCCTTCT |
| PolyC-1-NGS-8-F | CACCGGACTGCGGAAGTGAGGGGAGC |
| PolyC-1-NGS-8-R | ATCGTGTATCCGTGCGCGTAATCCTTCT |
| PolyC-1-NGS-9-F | ATGAGCACTGCGGAAGTGAGGGGAGC |
| PolyC-1-NGS-9-R | AGGAATTATCCGTGCGCGTAATCCTTCT |
| PolyC-1-NGS-10-F | CAAAAGACTGCGGAAGTGAGGGGAGC |
| PolyC-1-NGS-10-R | TAGTTGTATCCGTGCGCGTAATCCTTCT |
| PolyC-1-NGS-11-F | TCGGCAACTGCGGAAGTGAGGGGAGC |
| PolyC-1-NGS-11-R | GAATGATATCCGTGCGCGTAATCCTTCT |
| PolyC-1-NGS-12-F | TCCCGAACTGCGGAAGTGAGGGGAGC |
| PolyC-1-NGS-12-R | CTTCGATATCCGTGCGCGTAATCCTTCT |
| PolyC-2-NGS-1-F | ATCACGGACCTCGTTAGCTTTCTGTA |
| PolyC-2-NGS-1-R | GCCTAAACTTAATAGCTTGCCCTGTC |
| PolyC-2-NGS-2-F | CGATGTGACCTCGTTAGCTTTCTGTA |
| PolyC-2-NGS-2-R | TGGTCAACTTAATAGCTTGCCCTGTC |
| PolyC-2-NGS-3-F | AGTTCCGACCTCGTTAGCTTTCTGTA |
| PolyC-2-NGS-3-R | CTCTACACTTAATAGCTTGCCCTGTC |
| PolyC-2-NGS-4-F | CACTCAGACCTCGTTAGCTTTCTGTA |
| PolyC-2-NGS-4-R | TGTTGGACTTAATAGCTTGCCCTGTC |
| PolyC-2-NGS-5-F | GTGGCCGACCTCGTTAGCTTTCTGTA |
| PolyC-2-NGS-5-R | CGAAACACTTAATAGCTTGCCCTGTC |
| PolyC-2-NGS-6-F | CGTACGGACCTCGTTAGCTTTCTGTA |
| PolyC-2-NGS-6-R | CCACTCACTTAATAGCTTGCCCTGTC |
| PolyC-2-NGS-7-F | GGTAGCGACCTCGTTAGCTTTCTGTA |
| PolyC-2-NGS-7-R | ATCAGTACTTAATAGCTTGCCCTGTC |
| PolyC-2-NGS-8-F | CACCGGGACCTCGTTAGCTTTCTGTA |
| PolyC-2-NGS-8-R | ATCGTGACTTAATAGCTTGCCCTGTC |
| PolyC-2-NGS-9-F | ATGAGCGACCTCGTTAGCTTTCTGTA |
| PolyC-2-NGS-9-R | AGGAATACTTAATAGCTTGCCCTGTC |
| PolyC-2-NGS-10-F | CAAAAGGACCTCGTTAGCTTTCTGTA |
| PolyC-2-NGS-10-R | TAGTTGACTTAATAGCTTGCCCTGTC |
| PolyC-2-NGS-11-F | TCGGCAGACCTCGTTAGCTTTCTGTA |
| PolyC-2-NGS-11-R | GAATGAACTTAATAGCTTGCCCTGTC |
| PolyC-2-NGS-12-F | TCCCGAGACCTCGTTAGCTTTCTGTA |
| PolyC-2-NGS-12-R | CTTCGAACTTAATAGCTTGCCCTGTC |
| PolyC-3-NGS-1-F | ATCACGGTTTAGAACCTCGCTTTCCA |
| PolyC-3-NGS-1-R | GCCTAAAATGGATCTCTTTACTCTGGC |
| PolyC-3-NGS-2-F | CGATGTGTTTAGAACCTCGCTTTCCA |
| PolyC-3-NGS-2-R | TGGTCAAATGGATCTCTTTACTCTGGC |
| PolyC-3-NGS-3-F | AGTTCCGTTTAGAACCTCGCTTTCCA |
| PolyC-3-NGS-3-R | CTCTACAATGGATCTCTTTACTCTGGC |
| PolyC-3-NGS-4-F | CACTCAGTTTAGAACCTCGCTTTCCA |
| PolyC-3-NGS-4-R | TGTTGGAATGGATCTCTTTACTCTGGC |
| PolyC-3-NGS-5-F | GTGGCCGTTTAGAACCTCGCTTTCCA |
| PolyC-3-NGS-5-R | CGAAACAATGGATCTCTTTACTCTGGC |
| PolyC-3-NGS-6-F | CGTACGGTTTAGAACCTCGCTTTCCA |
| PolyC-3-NGS-6-R | CCACTCAATGGATCTCTTTACTCTGGC |
| PolyC-3-NGS-7-F | GGTAGCGTTTAGAACCTCGCTTTCCA |
| PolyC-3-NGS-7-R | ATCAGTAATGGATCTCTTTACTCTGGC |
| PolyC-3-NGS-8-F | CACCGGGTTTAGAACCTCGCTTTCCA |
| PolyC-3-NGS-8-R | ATCGTGAATGGATCTCTTTACTCTGGC |
| PolyC-3-NGS-9-F | ATGAGCGTTTAGAACCTCGCTTTCCA |
| PolyC-3-NGS-9-R | AGGAATAATGGATCTCTTTACTCTGGC |
| PolyC-3-NGS-10-F | CAAAAGGTTTAGAACCTCGCTTTCCA |
| PolyC-3-NGS-10-R | TAGTTGAATGGATCTCTTTACTCTGGC |
| PolyC-3-NGS-11-F | TCGGCAGTTTAGAACCTCGCTTTCCA |
| PolyC-3-NGS-11-R | GAATGAAATGGATCTCTTTACTCTGGC |
| PolyC-3-NGS-12-F | TCCCGAGTTTAGAACCTCGCTTTCCA |
| PolyC-3-NGS-12-R | CTTCGAAATGGATCTCTTTACTCTGGC |
| CAN1-1-NGS-1-F | TCATTCCAAAGAGGAAACCCAACCTA |
| CAN1-1-NGS-1-R | CTAGCTTGAACTTGTTCCCTGTCAAA |
| CAN1-1-NGS-2-F | GACGACCAAAGAGGAAACCCAACCTA |
| CAN1-1-NGS-2-R | TATAATTGAACTTGTTCCCTGTCAAA |
| CAN1-1-NGS-3-F | CTCAGACAAAGAGGAAACCCAACCTA |
| CAN1-1-NGS-3-R | TACAGCTGAACTTGTTCCCTGTCAAA |
| CAN1-2-NGS-1-F | TCGAAGTGGGTGAAATGGCTACATTC |
| CAN1-2-NGS-1-R | TAATCGACCGTAATATTTGACAGGGAA |
| CAN1-2-NGS-2-F | ATAAGTTGGGTGAAATGGCTACATTC |
| CAN1-2-NGS-2-R | GCTCAAACCGTAATATTTGACAGGGAA |
| CAN1-2-NGS-3-F | GCTATCTGGGTGAAATGGCTACATTC |
| CAN1-2-NGS-3-R | ATCCGAACCGTAATATTTGACAGGGAA |
| CAN1-3-NGS-1-F | CGTACCAAGCTTCACAAACACACCA |
| CAN1-3-NGS-1-R | GTCCGATTGGTCAGAGGTGTGGATAA |
| CAN1-3-NGS-2-F | GGTAGGAAGCTTCACAAACACACCA |
| CAN1-3-NGS-2-R | GTGGCGTTGGTCAGAGGTGTGGATAA |
| CAN1-3-NGS-3-F | CGATGAAAGCTTCACAAACACACCA |
| CAN1-3-NGS-3-R | CGTTCGTTGGTCAGAGGTGTGGATAA |
| CAN1-4-NGS-1-F | TGACCTTCGGTGTATGACTTATGAGG |
| CAN1-4-NGS-1-R | GGTTGCCTGCCGCCTATATCTCTATT |
| CAN1-4-NGS-2-F | ACATGATCGGTGTATGACTTATGAGG |
| CAN1-4-NGS-2-R | ATAAGCCTGCCGCCTATATCTCTATT |
| CAN1-4-NGS-3-F | TAGCTATCGGTGTATGACTTATGAGG |
| CAN1-4-NGS-3-R | GAAAACCTGCCGCCTATATCTCTATT |
| CAN1-5-NGS-1-F | CTTGTAGTCAAGGACCACCAAAGGTG |
| CAN1-5-NGS-1-R | CATTTTAGCCTGCAACACCAGTGATA |
| CAN1-5-NGS-2-F | GACGACGTCAAGGACCACCAAAGGTG |
| CAN1-5-NGS-2-R | TACAGCAGCCTGCAACACCAGTGATA |
| site-1-NGS-1-F | ATCACGAAATGCGCTCTTACTCGTAT |
| site-1-NGS-1-R | CGATGTAAGGGCTAAAGTATCAGAGC |
| site-1-NGS-2-F | ACTGATAAATGCGCTCTTACTCGTAT |
| site-1-NGS-2-R | CAGGCGAAGGGCTAAAGTATCAGAGC |
| site-1-NGS-3-F | TAGTTGAAATGCGCTCTTACTCGTAT |
| site-1-NGS-3-R | GAATGAAAGGGCTAAAGTATCAGAGC |
| site-1-NGS-4-F | CACGATAAATGCGCTCTTACTCGTAT |
| site-1-NGS-4-R | CATTTTAAGGGCTAAAGTATCAGAGC |
| site-2-NGS-1-F | AGTTCCCACTGTCGGTGTAAATTTGG |
| site-2-NGS-1-R | CACTCATGGTCATTCGATCACAACAT |
| site-2-NGS-2-F | ACAGTGCACTGTCGGTGTAAATTTGG |
| site-2-NGS-2-R | CAACTATGGTCATTCGATCACAACAT |
| site-2-NGS-3-F | CTTCGACACTGTCGGTGTAAATTTGG |
| site-2-NGS-3-R | TCTGAGTGGTCATTCGATCACAACAT |
| site-2-NGS-4-F | CGGAATCACTGTCGGTGTAAATTTGG |
| site-2-NGS-4-R | ATAAGTTGGTCATTCGATCACAACAT |
| site-3-NGS-1-F | GTGGCCCTCCTATGAAGCGGAACAG |
| site-3-NGS-1-R | CGTACCCGAGCAAGAATTCACCTCT |
| site-3-NGS-2-F | CTTGCACTCCTATGAAGCGGAACAG |
| site-3-NGS-2-R | ATGTCACGAGCAAGAATTCACCTCT |
| site-3-NGS-3-F | TGACCACTCCTATGAAGCGGAACAG |
| site-3-NGS-3-R | CATGGCCGAGCAAGAATTCACCTCT |
| site-3-NGS-4-F | CGATGACTCCTATGAAGCGGAACAG |
| site-3-NGS-4-R | TGACCTCGAGCAAGAATTCACCTCT |
| site-4-NGS-1-F | GGTAGGGTTTGAACAACATTCAGGCT |
| site-4-NGS-1-R | CACCGGTCGACAACTCTACCCAAAAG |
| site-4-NGS-2-F | CCAACAGTTTGAACAACATTCAGGCT |
| site-4-NGS-2-R | TCATTCTCGACAACTCTACCCAAAAG |
| site-4-NGS-3-F | GCCAATGTTTGAACAACATTCAGGCT |
| site-4-NGS-3-R | TATAATTCGACAACTCTACCCAAAAG |
| site-4-NGS-4-F | ACATGAGTTTGAACAACATTCAGGCT |
| site-4-NGS-4-R | TAGCTATCGACAACTCTACCCAAAAG |
| site-5-NGS-1-F | ATGAGCTCATCCGCAGCTAATTTTGA |
| site-5-NGS-1-R | CAAAAGTTCGAATACGCTCGCAAATA |
| site-5-NGS-2-F | TCGAAGTCATCCGCAGCTAATTTTGA |
| site-5-NGS-2-R | GACGACTTCGAATACGCTCGCAAATA |
| site-5-NGS-3-F | CTAGCTTCATCCGCAGCTAATTTTGA |
| site-5-NGS-3-R | CTCAGATTCGAATACGCTCGCAAATA |
| site-5-NGS-4-F | CTAGTATCATCCGCAGCTAATTTTGA |
| site-5-NGS-4-R | AGTTCATTCGAATACGCTCGCAAATA |
| site-6-NGS-1-F | TCGGCAACTTACGAGGTTTTCCAAGG |
| site-6-NGS-1-R | TCCCGATCCTTCGGATTCAAGCATTT |
| site-6-NGS-2-F | TACAGCACTTACGAGGTTTTCCAAGG |
| site-6-NGS-2-R | GCCTAATCCTTCGGATTCAAGCATTT |
| site-6-NGS-3-F | TAATCGACTTACGAGGTTTTCCAAGG |
| site-6-NGS-3-R | ATCACCTCCTTCGGATTCAAGCATTT |
| site-6-NGS-4-F | CCGTCAACTTACGAGGTTTTCCAAGG |
| site-6-NGS-4-R | GTCCGATCCTTCGGATTCAAGCATTT |
| site-7-NGS-1-F | CTATACTTATCGCGGTCTTACTTACG |
| site-7-NGS-1-R | TTAGGCCAATTAATCACCCGTTCTGC |
| site-7-NGS-2-F | TGGTCATTATCGCGGTCTTACTTACG |
| site-7-NGS-2-R | CTCTACCAATTAATCACCCGTTCTGC |
| site-7-NGS-3-F | GTAGAGTTATCGCGGTCTTACTTACG |
| site-7-NGS-3-R | GTGAAACAATTAATCACCCGTTCTGC |
| site-7-NGS-4-F | GTGGCGTTATCGCGGTCTTACTTACG |
| site-7-NGS-4-R | CGTTCGCAATTAATCACCCGTTCTGC |
| site-8-NGS-1-F | TAGCTTTGTTACCTGGCAGATCCTTA |
| site-8-NGS-1-R | CCGTCCCACCAAAGTACGTTCAGAGA |
| site-8-NGS-2-F | CCACTCTGTTACCTGGCAGATCCTTA |
| site-8-NGS-2-R | ATCAGTCACCAAAGTACGTTCAGAGA |
| site-8-NGS-3-F | GGCTACTGTTACCTGGCAGATCCTTA |
| site-8-NGS-3-R | AGTCAACACCAAAGTACGTTCAGAGA |
| site-8-NGS-4-F | GAAAACTGTTACCTGGCAGATCCTTA |
| site-8-NGS-4-R | CAAAGGCACCAAAGTACGTTCAGAGA |
| site-9-NGS-1-F | GTCCGCGTTACCGACATTGGAGGATT |
| site-9-NGS-1-R | GTTTCGGGATTTCTGAGAGGTATGGG |
| site-9-NGS-2-F | ATCGTGGTTACCGACATTGGAGGATT |
| site-9-NGS-2-R | AGGAATGGATTTCTGAGAGGTATGGG |
| site-9-NGS-3-F | GAGTGGGTTACCGACATTGGAGGATT |
| site-9-NGS-3-R | ATTCCTGGATTTCTGAGAGGTATGGG |
| site-9-NGS-4-F | CACTCTGTTACCGACATTGGAGGATT |
| site-9-NGS-4-R | CATGGAGGATTTCTGAGAGGTATGGG |
| site-10-NGS-1-F | ATCACGATTCATCTACTCGTTGGTCG |
| site-10-NGS-1-R | CGATGTCAGTTATTGAAAACGGCGAA |
| site-10-NGS-2-F | AGTTCCATTCATCTACTCGTTGGTCG |
| site-10-NGS-2-R | CACTCACAGTTATTGAAAACGGCGAA |
| site-10-NGS-3-F | GTGGCCATTCATCTACTCGTTGGTCG |
| site-10-NGS-3-R | CGTACCCAGTTATTGAAAACGGCGAA |
| site-10-NGS-4-F | GGTAGGATTCATCTACTCGTTGGTCG |
| site-10-NGS-4-R | CACCGGCAGTTATTGAAAACGGCGAA |
| site-11-NGS-1-F | CTTGCAGTTCCGAAACAGGACACTTA |
| site-11-NGS-1-R | ATGTCAAAGTGGTCTGTCGAAAGTAG |
| site-11-NGS-2-F | CCAACAGTTCCGAAACAGGACACTTA |
| site-11-NGS-2-R | TCATTCAAGTGGTCTGTCGAAAGTAG |
| site-11-NGS-3-F | TCGAAGGTTCCGAAACAGGACACTTA |
| site-11-NGS-3-R | GACGACAAGTGGTCTGTCGAAAGTAG |
| site-11-NGS-4-F | TACAGCGTTCCGAAACAGGACACTTA |
| site-11-NGS-4-R | GCCTAAAAGTGGTCTGTCGAAAGTAG |
| site-12-NGS-1-F | CTAGCTTTGCGATCATCACCGTTAAA |
| site-12-NGS-1-R | CTCAGACGCTCAAATGATCGATACCA |
| site-12-NGS-2-F | TAATCGTTGCGATCATCACCGTTAAA |
| site-12-NGS-2-R | ATCACCCGCTCAAATGATCGATACCA |
| site-12-NGS-3-F | GTAGAGTTGCGATCATCACCGTTAAA |
| site-12-NGS-3-R | GTGAAACGCTCAAATGATCGATACCA |
| site-12-NGS-4-F | CAGATCTTGCGATCATCACCGTTAAA |
| site-12-NGS-4-R | GATCAGCGCTCAAATGATCGATACCA |
| site-13-NGS-1-F | ATCACGATTTACTCTCGTCGGGAAAG |
| site-13-NGS-1-R | CGATGTAAATTCAAAAGAAGACGCCG |
| site-13-NGS-2-F | AGTTCCATTTACTCTCGTCGGGAAAG |
| site-13-NGS-2-R | CACTCAAAATTCAAAAGAAGACGCCG |
| site-13-NGS-3-F | GTGGCCATTTACTCTCGTCGGGAAAG |
| site-13-NGS-3-R | CGTACCAAATTCAAAAGAAGACGCCG |
| site-13-NGS-4-F | GGTAGGATTTACTCTCGTCGGGAAAG |
| site-13-NGS-4-R | CACCGGAAATTCAAAAGAAGACGCCG |
| site-14-NGS-1-F | CTTGCATAGAACTGTTGATTTGCGGT |
| site-14-NGS-1-R | ATGTCACAGGTGAAAATGTTGCTGAG |
| site-14-NGS-2-F | CCAACATAGAACTGTTGATTTGCGGT |
| site-14-NGS-2-R | TCATTCCAGGTGAAAATGTTGCTGAG |
| site-14-NGS-3-F | TCGAAGTAGAACTGTTGATTTGCGGT |
| site-14-NGS-3-R | GACGACCAGGTGAAAATGTTGCTGAG |
| site-14-NGS-4-F | TACAGCTAGAACTGTTGATTTGCGGT |
| site-14-NGS-4-R | GCCTAACAGGTGAAAATGTTGCTGAG |
| site-15-NGS-1-F | CTAGCTTTAGTTTTGAGCCACAATGC |
| site-15-NGS-1-R | CTCAGAGTCCGATTACAGCCGATTAT |
| site-15-NGS-2-F | TAATCGTTAGTTTTGAGCCACAATGC |
| site-15-NGS-2-R | ATCACCGTCCGATTACAGCCGATTAT |
| site-15-NGS-3-F | GTAGAGTTAGTTTTGAGCCACAATGC |
| site-15-NGS-3-R | GTGAAAGTCCGATTACAGCCGATTAT |
| site-15-NGS-4-F | CAGATCTTAGTTTTGAGCCACAATGC |
| site-15-NGS-4-R | GATCAGGTCCGATTACAGCCGATTAT |
| site-16-NGS-1-F | AGTTCCATAACGACAAATACAGGCCC |
| site-16-NGS-1-R | CTCTACTCGGCAAGTACTAAACAGAG |
| site-16-NGS-2-F | CACTCAATAACGACAAATACAGGCCC |
| site-16-NGS-2-R | TGTTGGTCGGCAAGTACTAAACAGAG |
| site-17-NGS-1-F | GTGGCCCTGAGCTACCAAATCAGTCT |
| site-17-NGS-1-R | CGAAACTTTCCTCTCTTTTCCCGAAG |
| site-17-NGS-2-F | CGTACGCTGAGCTACCAAATCAGTCT |
| site-17-NGS-2-R | CCACTCTTTCCTCTCTTTTCCCGAAG |
| site-18-NGS-1-F | GGTAGCAAACCCCATTTGGAAGGAAT |
| site-18-NGS-1-R | ATCAGTAGGTGGAATATCTCTTATGCC |
| site-18-NGS-2-F | CACCGGAAACCCCATTTGGAAGGAAT |
| site-18-NGS-2-R | ATCGTGAGGTGGAATATCTCTTATGCC |
| site-19-NGS-1-F | TCGGCAGAATAAACATACGCAAGGCG |
| site-19-NGS-1-R | GAATGACAAACTGCAGATGTGTTTGA |
| site-19-NGS-2-F | TCCCGAGAATAAACATACGCAAGGCG |
| site-19-NGS-2-R | CTTCGACAAACTGCAGATGTGTTTGA |
| site-20-NGS-1-F | CTATACCCAACCTCTTTTCAGGATCT |
| site-20-NGS-1-R | TCTGAGGGTGCTGCTATATCAAATGC |
| site-20-NGS-2-F | TTAGGCCCAACCTCTTTTCAGGATCT |
| site-20-NGS-2-R | TGACCAGGTGCTGCTATATCAAATGC |
| site-21-NGS-1-F | ACATGTATCAGTCTCTACTCAGCCAA |
| site-21-NGS-1-R | CAGATCCTATCTTCATTACCCAGCCC |
| site-21-NGS-2-F | ACTTGAATCAGTCTCTACTCAGCCAA |
| site-21-NGS-2-R | GATCAGCTATCTTCATTACCCAGCCC |
| site-22-NGS-1-F | TAGCTTTGAATATGAGTTCGTCGCAT |
| site-22-NGS-1-R | GGCTACCGTACTCTATGACATCGACC |
| site-22-NGS-2-F | CCGTCCTGAATATGAGTTCGTCGCAT |
| site-22-NGS-2-R | GTAGAGCGTACTCTATGACATCGACC |
| site-23-NGS-1-F | GTCCGCGAGACAGTCTTTGACATGCT |
| site-23-NGS-1-R | GTGAAAGAAAGGCTTCTGCACAATTT |
| site-23-NGS-2-F | GTTTCGGAGACAGTCTTTGACATGCT |
| site-23-NGS-2-R | GAGTGGGAAAGGCTTCTGCACAATTT |
| site-24-NGS-1-F | ACTGATGGTAGAAGAAGCAGAGCATC |
| site-24-NGS-1-R | ATTCCTATACTTTACGCAAGAGGGTC |
| site-24-NGS-2-F | CAACTAGGTAGAAGAAGCAGAGCATC |
| site-24-NGS-2-R | CACGATATACTTTACGCAAGAGGGTC |
| site-25-NGS-1-F | CAGGCGTAATTGGGATAACTGGCGTG |
| site-25-NGS-1-R | CATGGCTTTCCAAGACAACGGATGAT |
| site-25-NGS-2-F | ACAGTGTAATTGGGATAACTGGCGTG |
| site-25-NGS-2-R | GCCAATTTTCCAAGACAACGGATGAT |
| site-26-NGS-1-F | CTTGTATCTACCACTTTTCCCAGGAT |
| site-26-NGS-1-R | AGTCAATATTTACCTCCCGAACAACG |
| site-26-NGS-2-F | ATGTCATCTACCACTTTTCCCAGGAT |
| site-26-NGS-2-R | CATTTTTATTTACCTCCCGAACAACG |
| site-27-NGS-1-F | CCAACAACAGGTCTTGTAAAGCCATT |
| site-27-NGS-1-R | CGGAATCTTGTATCCGGACTGAACTC |
| site-27-NGS-2-F | TCATTCACAGGTCTTGTAAAGCCATT |
| site-27-NGS-2-R | CTAGCTCTTGTATCCGGACTGAACTC |
| site-28-NGS-1-F | TCGAAGCTAAGGTCATCTCCATCTGC |
| site-28-NGS-1-R | TAATCGCGAAGTTGGTATCCTCACAT |
| site-28-NGS-2-F | ATAAGTCTAAGGTCATCTCCATCTGC |
| site-28-NGS-2-R | GCTCAACGAAGTTGGTATCCTCACAT |
| site-29-NGS-1-F | GCTATCAAGAGGGCTAAAAGAAGCTTCAGTG |
| site-29-NGS-1-R | ATCCGATATTGGGCTTACGGGACT |
| site-29-NGS-2-F | CGTACCAAGAGGGCTAAAAGAAGCTTCAGTG |
| site-29-NGS-2-R | GTCCGATATTGGGCTTACGGGACT |
| site-30-NGS-1-F | GGTAGGCTCTTGGATTGTACCCGTAA |
| site-30-NGS-1-R | GTGGCGACTGTGTTGCTGATAGATGG |
| site-30-NGS-2-F | CGATGACTCTTGGATTGTACCCGTAA |
| site-30-NGS-2-R | CGTTCGACTGTGTTGCTGATAGATGG |
| site-31-NGS-1-F | TGACCTAAATTGCCATAACCAGTTGC |
| site-31-NGS-1-R | GGTTGCAGGTAATGCCATGATTGTGT |
| site-31-NGS-2-F | ACATGAAAATTGCCATAACCAGTTGC |
| site-31-NGS-2-R | ATAAGCAGGTAATGCCATGATTGTGT |
| site-32-NGS-1-F | TAGCTATCAATTGACCCACATGGAAG |
| site-32-NGS-1-R | GAAAACTAATTTCAACCAGAGCAAAGC |
| site-32-NGS-2-F | CTAGTATCAATTGACCCACATGGAAG |
| site-32-NGS-2-R | CAAAGGTAATTTCAACCAGAGCAAAGC |
| site-33-NGS-1-F | GATCATTTTCAGAAGCAGCTTTACCT |
| site-33-NGS-1-R | CCTAGTAAGAACAAATACTGGTCCCC |
| site-33-NGS-2-F | AGCTAGTTTCAGAAGCAGCTTTACCT |
| site-33-NGS-2-R | GATCTAAAGAACAAATACTGGTCCCC |
| site-34-NGS-1-F | ATCACGCAATCATGTCACAGTACGGA |
| site-34-NGS-1-R | GCCTAAGCAAAGAGTGGTTTAGAACG |
| site-34-NGS-2-F | CGATGTCAATCATGTCACAGTACGGA |
| site-34-NGS-2-R | TGGTCAGCAAAGAGTGGTTTAGAACG |
| site-35-NGS-1-F | ATGAGCAATTGGATCTGGTAAGGTCG |
| site-35-NGS-1-R | GAATGATTACCCTTTAGGGACTGGAA |
| site-35-NGS-2-F | CTATACAATTGGATCTGGTAAGGTCG |
| site-35-NGS-2-R | CAGATCTTACCCTTTAGGGACTGGAA |
| site-36-NGS-1-F | TAGCTTATGTCTCTCTTTAGCATCGC |
| site-36-NGS-1-R | GGCTACTAGAAAGCTGCTTAGTTGGG |
| site-36-NGS-2-F | CAACTAATGTCTCTCTTTAGCATCGC |
| site-36-NGS-2-R | CACGATTAGAAAGCTGCTTAGTTGGG |
| *OsALS*-NGS-F | AGAGGGAGTTTCCTCTGGGGT |
| *OsALS*-NGS-R | CCACATCTGGTGCTGCCCAA |
| *OsCDC48*-NGS-F | TGCTAACGAGTGCCAGGCTAAC |
| *OsCDC48*-NGS-R | CACTGCTTCCTCTCTGGGTAGCAA |
| *OsGS1*-NGS-F | CCTGTGACTGATCCCAGCAAG |
| *OsGS1*-NGS-R | CTTGAAGATAGCCTGTGGGC |
| *OsSLR1*-NGS-F | CGGATGACGGGTTCGTGT |
| *OsSLR1*-NGS-R | GTTCAAAGAAGCCGCTACCA |


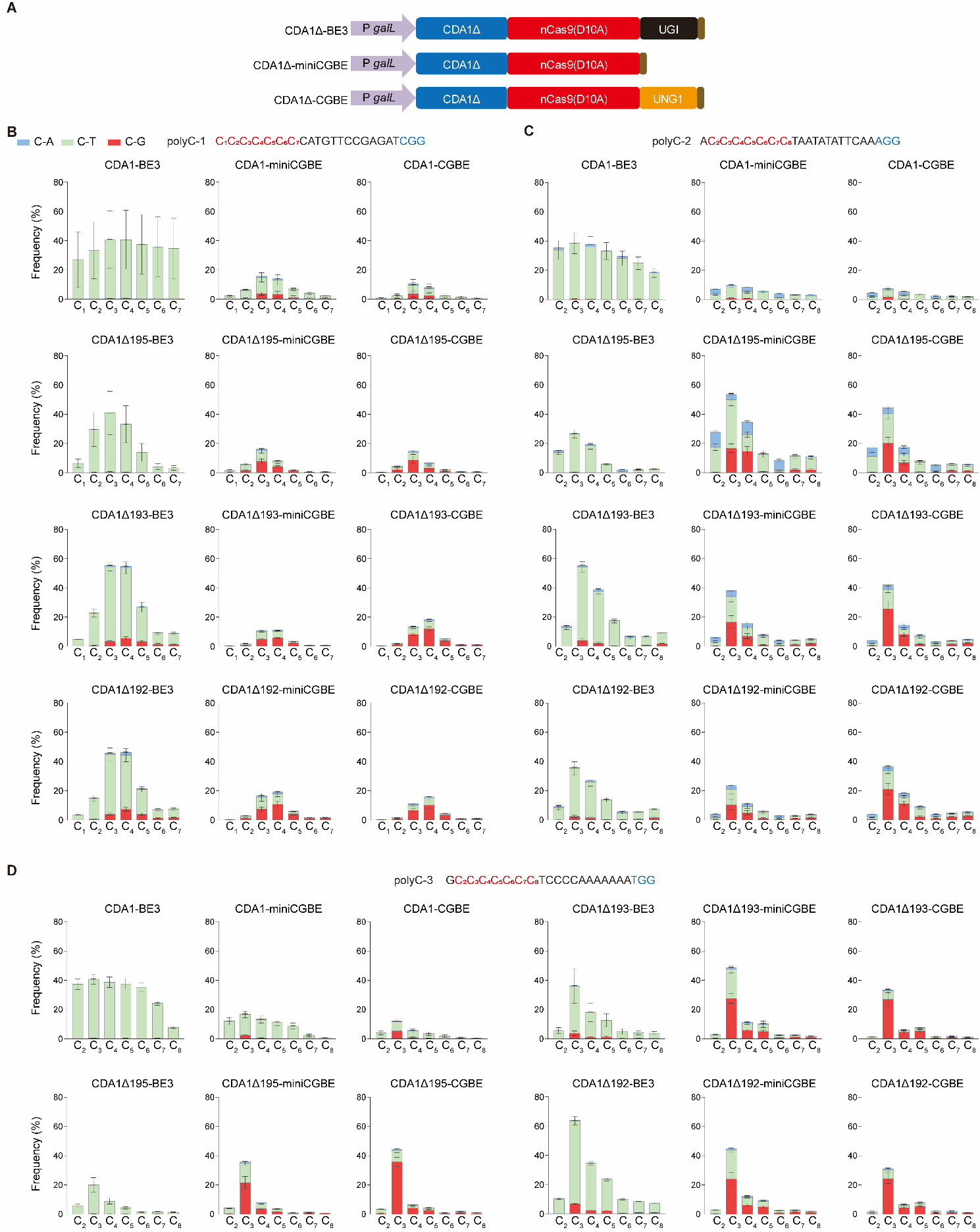


**Figure S1. Effects of C-terminal truncations of the CDA1 domain on the efficiency of C-to-G base editing of CDA1-derived CGBEs at three polyC-containing target sites.**

**(A)** Design of the CBE, miniCGBE, and CGBE constructs with C-terminally truncated CDA1 variants. **(B-D)** Comparison of on-target base editing frequencies of CBEs, miniCGBEs, and CGBEs with CDA1 truncations, determined at the three polyC-containing target sites. Cs within each target region are highlighted in red, with the number below indicating their distance from the distal end of the PAM (blue). Values and error bars represent the mean and standard deviation of three biological replicates. Other variants are shown in Figure 2. Source data is provided as a Source Data file.


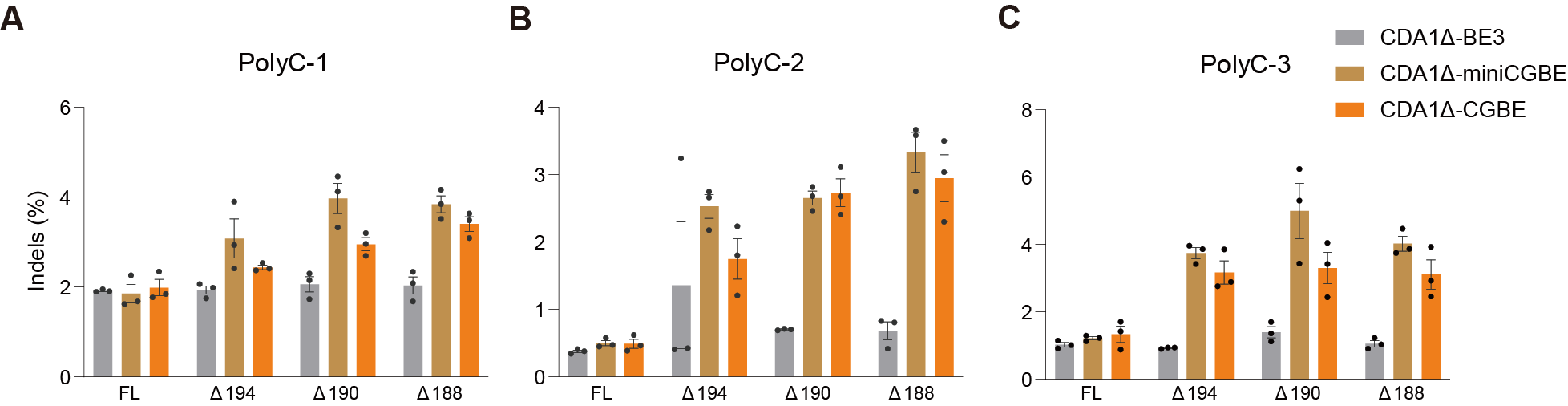


**Figure S2. Comparison of the frequency of indel formation among truncated CDA1-based BE variants.**

**(A-C)** Graphs showing indel mutation frequencies for CBEs, miniCGBEs, and CGBEs with truncated CDA1 domains across the three polyC-containing sites shown in Figure 2. Values and error bars represent the mean and standard deviation of three biological replicates. Source data is provided as a Source Data file.

**
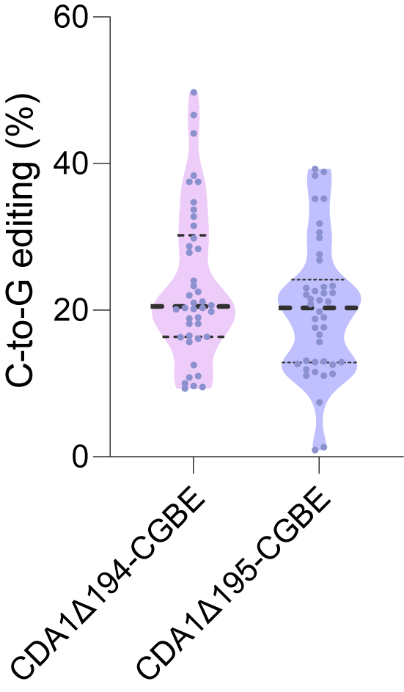
Figure S3. Comparison of C-to-G editing efficiency between CDA1Δ194-CGBE and CDA1Δ195-CGBE**

C-to-G editing efficiencies at the C_3_ position of 15 target sites were compared between CDA1Δ194-CGBE (pink) and CDA1Δ195-CGBE (blue). Each dot represents the editing efficiency of an individual biological replicate. The thick black dashed line indicates the average editing efficiency for each construct, while the thin black dashed lines represent the quartiles of the data distribution. Source data is provided as a Source Data file.

**
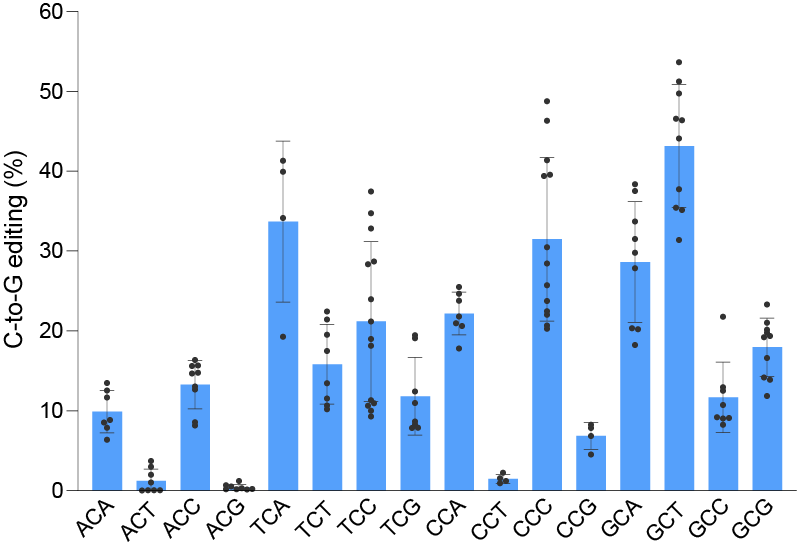
**

**Figure S4. Evaluation of C-to-G editing efficiency of CDA1Δ194-CGBE at each NC_3_N DNA motif.**

A total of 37 different gRNAs were designed to target the corresponding genomic region in yeast (*Can1-5*; CGBE site 1 to CGBE site 36 in Supplemental Table 1). Each dot represents the C-to-G editing at C_3_ for an individual biological replicate, with values and error bars indicating the mean and standard deviation across all biological replicates. Source data is provided as a Source Data file.

**
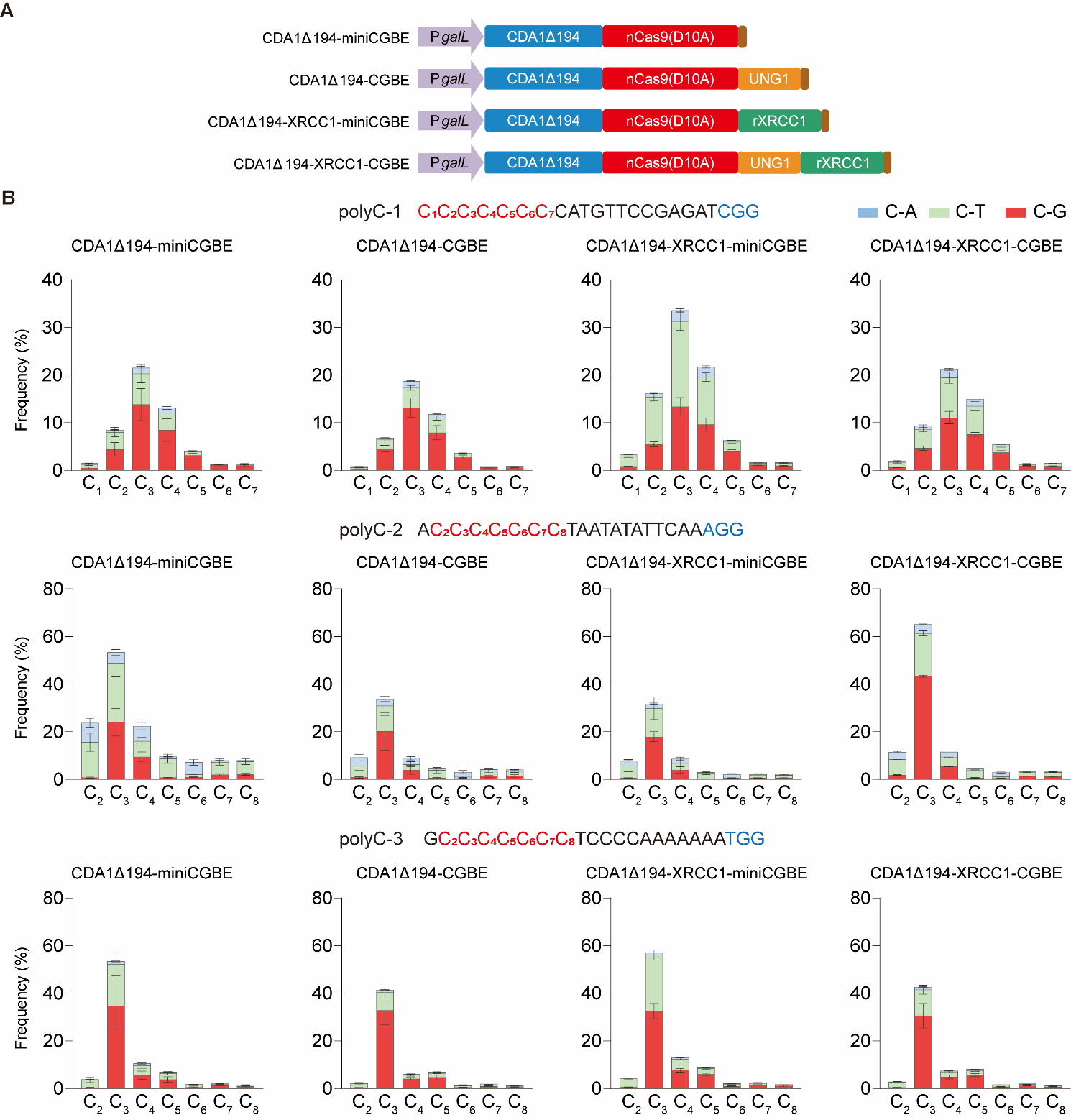
**

**Figure S5. Effects of XRCC1 fusion on the efficiency of base editing in CDA1Δ194-based CGBEs.**

**(A)** Illustration of the structures of CDA1Δ194-based CGBEs and their fusions with XRCC1. XRCC1: X-ray repair cross-complementing protein 1, a DNA repair protein from *Rattus norvegicus*. **(B)** Comparison of on-target base editing frequencies for CDA1Δ194-based CGBEs with or without the XRCC1 fusion at three polyC-containing sites. Values and error bars represent the mean and standard deviation of three biological replicates. Source data is provided as a Source Data file.


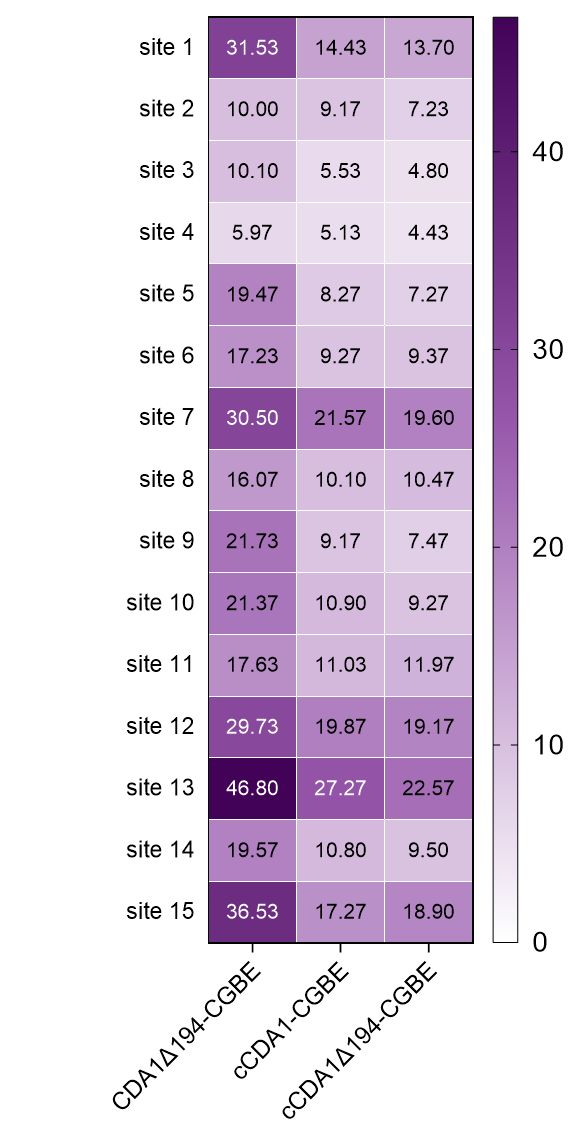
**Figure S6. Comparison of C-to-G efficiencies of CDA1Δ194-CGBE, cCDA1-CGBE and cCDA1Δ194-CGBE.**

Heatmaps showing C-to-G editing frequencies at the C_3_ position across fifteen target sites for CDA1Δ194-CGBE, cCDA1-CGBE and cCDA1Δ194-CGBE, with color gradients ranging from light purple (indicating low editing frequency) to dark purple (indicating high editing frequency). Values inside each cell indicate the mean editing frequency (in %) of three independent biological replicates at the corresponding cytosine position. Source data underlying this figure is provided as a Source Data file.

**
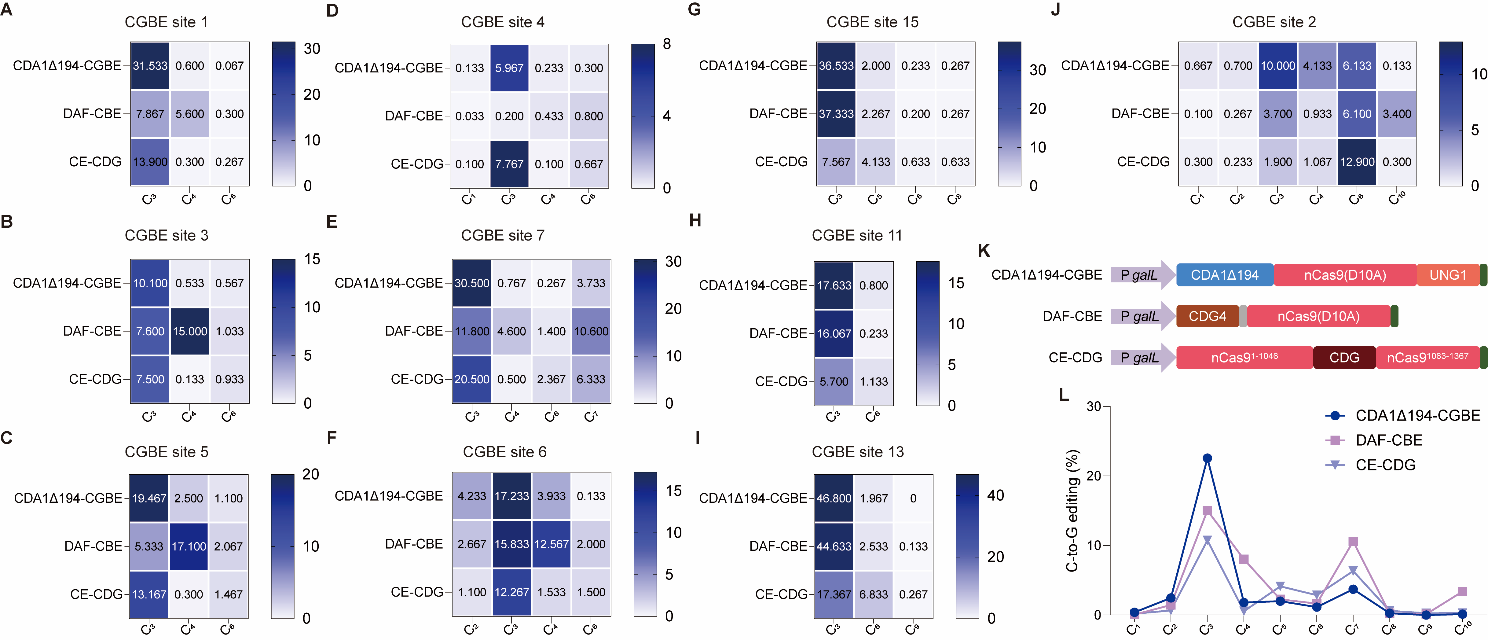
**

**Figure S7. Comparison of editing efficiencies between CDA1Δ194-CGBE, DAF-CBE, and CE-CDG.**

**(A-J)** Heatmaps displaying C-to-G editing frequencies at various cytosine positions across ten target sites. Each heatmap represents the editing frequency for specific cytosine positions within the target sequences, with color gradients ranging from light blue (indicating low editing frequency) to dark blue (indicating high editing frequency). Values inside each cell indicate the mean editing frequency (in %) of three independent biological replicates at the corresponding cytosine position. **(K)** Schematic representation of the structural configurations of the three CGBEs. The gray box indicates the linker, while the brown box represents the NLS. **(L)** Mean C-to-G editing efficiency across position 1-10 for each BE. Each point represents the mean value of three independent experiments for each of the ten target sites. Source data for **(A-J)** and **(L)** are provided as a Source Data file.

**
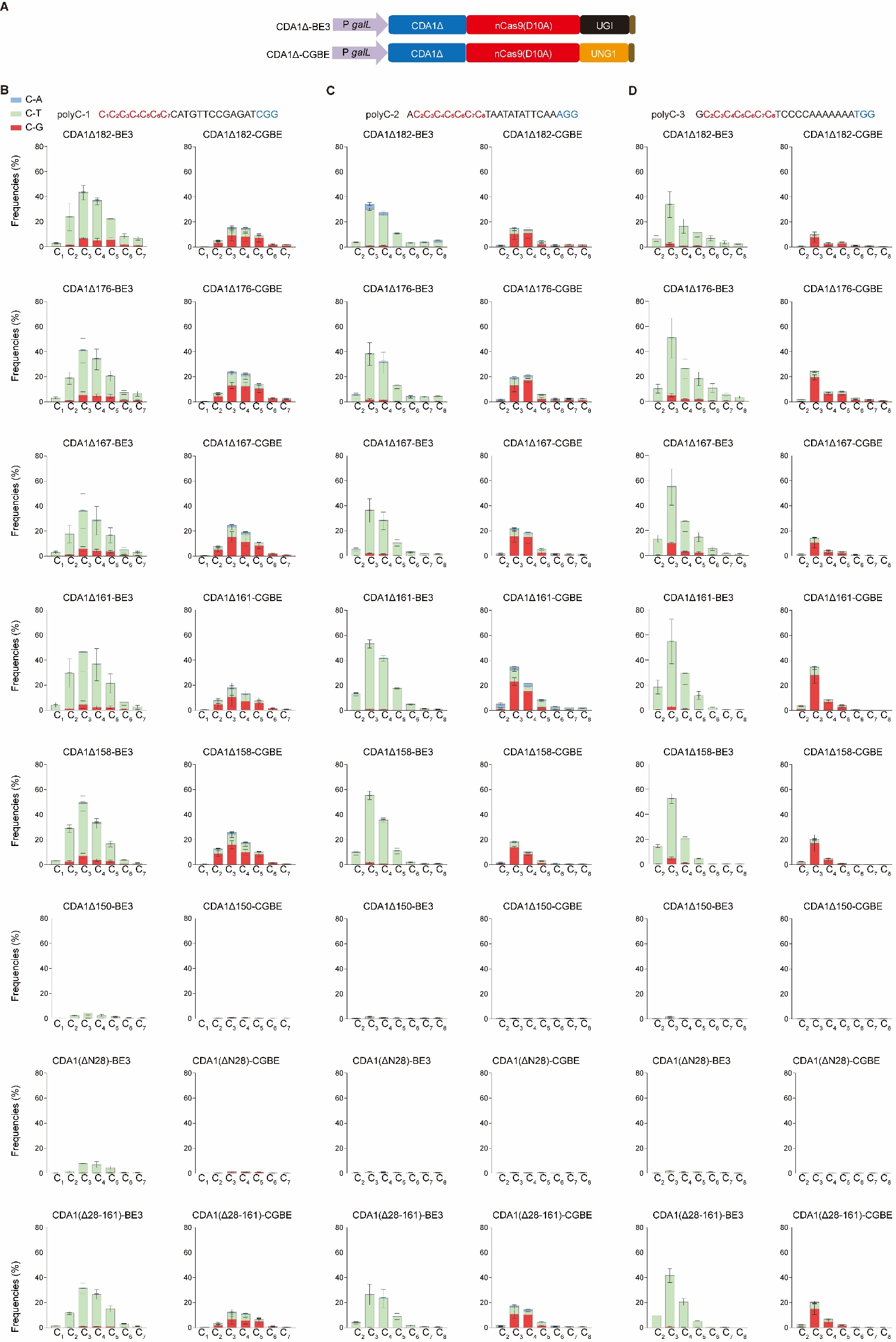
**

**Figure S8. Effects of larger C-terminal deletions of the CDA1 domain on the efficiency of C-to-G base editing of CDA1-derived CGBEs at the three polyC-containing target sites.**

**(A)** Schematic illustration of the structures of the CBEs and CGBEs harboring truncated CDA1 domains. **(B-D)** Comparative analysis of on-target base editing frequencies in CBEs and CGBEs with larger C-terminal CDA1 truncations (Δ182 aa to Δ135 aa) at the three polyC-containing sites. Values and error bars represent the mean and standard deviation of three biological replicates. For abbreviations and additional information, see Figure 2 and Supplementary Figure 1. Source data is provided as a Source Data file.


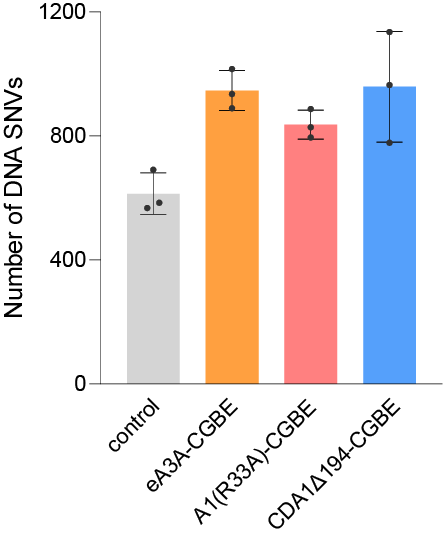


**Figure S9.** **Comparison of off-target DNA editing between** **eA3A-CGBE, A1(R33A)-CGBE, and CDA1Δ194-CGBE.**

The sgRNA used was designed to target site *Can1-5* for CDA1Δ194-CGBE and *Can1-6* for eA3A-CGBE and A1(R33A)-CGBE (Supplementary Table 1). Values and error bars represent the mean and standard deviation of three independent biological replicates. Source data is provided as a Source Data file.


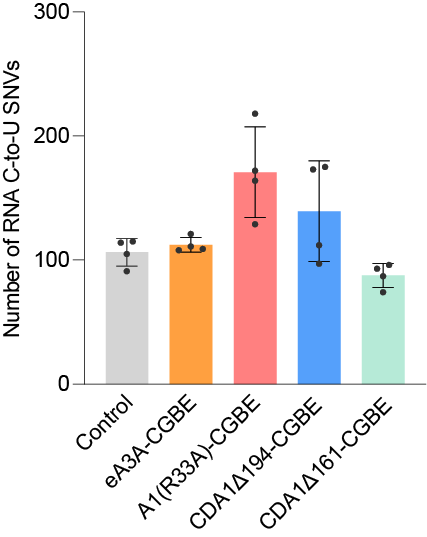


**Figure S10. Transcriptome-wide analysis of off-target RNA editing.**

Comparison of the number of C-to-U single nucleotide variants (SNVs) in cellular transcripts among groups treated with different CGBEs, including eA3A-CGBE, A1(R33A)-CGBE, and two CGBEs with CDA1 truncations (CDA1Δ194-CGBE and CDA1Δ161-CGBE), along with the control group. Values and error bars represent the mean and standard deviation of four biological replicates. Source data is provided as a Source Data file.
